# Design, Synthesis and Comprehensive Investigations of Pyrrolo[3,4-*d*]pyridazinone-Based 1,3,4-Oxadiazole as New Class of Selective COX-2 Inhibitors

**DOI:** 10.3390/ijms21249623

**Published:** 2020-12-17

**Authors:** Łukasz Szczukowski, Edward Krzyżak, Adrianna Zborowska, Patrycja Zając, Katarzyna Potyrak, Krzysztof Peregrym, Benita Wiatrak, Aleksandra Marciniak, Piotr Świątek

**Affiliations:** 1 Department of Medicinal Chemistry, Wroclaw Medical University, Borowska 211, 50-556 Wroclaw, Poland; piotr.swiatek@umed.wroc.pl; 2 Department of Inorganic Chemistry, Wroclaw Medical University, Borowska 211a, 50-556 Wroclaw, Poland; edward.krzyzak@umed.wroc.pl (E.K.); aleksandra.marciniak@umed.wroc.pl (A.M.); 3 Student Scientific Club of Medicinal Chemistry, Wroclaw Medical University, Borowska 211, 50-556 Wroclaw, Poland; adrianna.zborowska@student.umed.wroc.pl (A.Z.); patrycja.zajac@student.umed.wroc.pl (P.Z.); katarzyna.potyrak@student.umed.wroc.pl (K.P.); krzysztof.peregrym@student.umed.wroc.pl (K.P.); 4 Department of Basic Medical Sciences, Wroclaw Medical University, Borowska 211, 50-556 Wroclaw, Poland; benita.wiatrak@umed.wroc.pl; 5 Department of Pharmacology, Wroclaw Medical University, Mikulicza-Radeckiego 2, 50-345 Wroclaw, Poland

**Keywords:** selective COX-2 inhibitors, double pharmacophore approach, anti-inflammatory activity, anti-oxidant agents, molecular docking, 1,3,4-oxadiazole, DNA protection

## Abstract

The long-term use of Non-Steroidal Anti-Inflammatory Drugs (NSAIDs) in treatment of different chronic inflammatory disorders is strongly restricted by their serious gastrointestinal adverse effects. Therefore, there is still an urgent need to search for new, safe, and efficient anti-inflammatory agents. Previously, we have reported the Mannich base-type derivatives of pyrrolo[3,4-*d*]pyridazinone which strongly inhibit cyclooxygenase, have better affinity to COX-2 isoenzyme and exert promising anti-oxidant activity. These findings encouraged us to perform further optimization of that structure. Herein, we present the design, synthesis, molecular docking, spectroscopic, and biological studies of novel pyrrolo[3,4-*d*]pyridazinone derivatives bearing 4-aryl-1-(1-oxoethyl)piperazine pharmacophore **5a,b**–**6a,b**. The new compounds were obtained via convenient, efficient, one-pot synthesis. According to in vitro evaluations, novel molecules exert no cytotoxicity and act as selective COX-2 inhibitors. These findings stay in good correlation with molecular modeling results, which additionally showed that investigated compounds take a position in the active site of COX-2 very similar to Meloxicam. Moreover, all derivatives reduce the increased level of reactive oxygen and nitrogen species and prevent DNA strand breaks caused by oxidative stress. Finally, performed spectroscopic and molecular docking studies demonstrated that new compound interactions with bovine serum albumin (BSA) are moderate, formation of complexes is in one-to-one ratio, and binding site II (subdomain IIIA) is favorable.

## 1. Introduction

The prostaglandin-endoperoxide synthase (PGH synthase), more widely known as cyclooxygenase (COX), is a membrane-bound enzyme that catalyzes the conversion of arachidonic acid into bioactive lipids such as prostaglandins (PGs) and thromboxane (TX) [1,2,3,4,5,6]. These mediators are essential to maintain homeostasis and play a crucial role in various pathological conditions such as induction of pain and control of inflammation. Cyclooxygenase exists in three isoforms that differ from each other in terms of structure and function [1,2,3,4]. A constitutive form of the enzyme, named COX-1, is expressed in normal cells. It synthesizes PGs involved in the physiological processes related mainly to the gastrointestinal and cardiovascular systems. For instance, activation of COX-1 in gastric mucosa leads to prostaglandin I_2_ (PGI_2_) formation, commonly called prostacyclin, which exerts cytoprotective effects by increasing secretion of mucus and bicarbonate and improving local blood flow. Prostacyclin produced in endothelial cells reduces platelet aggregation and causes vasodilation [1,2,3,4,5,6,7,8]. COX-2, on the contrary, is mostly an inducible form of cyclooxygenase, which level under normal, physiological conditions, is very low. Its expression immediately increases due to various pro-inflammatory and pathogenic stimuli. Induction of isoform COX-2 occurs in areas affected by inflammation, infection, neurodegeneration or cell mutation [1,2,3,9,10]. Finally, the third isoform named COX-3, a variant of COX-1, is expressed mainly in the brain and spinal cord [1,2,11].

Both cyclooxygenase isoforms, COX-1 and COX-2, are molecular targets for salicylates and aspirin-like drugs, more often called presently Non-steroidal and Anti-Inflammatory Drugs (NSAIDs). It was discovered in 1971 by Vane that popular antipyretic and anti-inflammatory drug—aspirin diminish prostanoid biosynthesis. Inhibition of COX expressed in malfunctioning tissue affected by injury or infection results in pain relief and reduction of inflammation. Unfortunately, at the same time, the decreased level of prostaglandins in normal cells causes dangerous side effects characteristic for NSAIDs [1,2,3,4,5,6,7,8,9,10,12,13,14].

Non-steroidal and Anti-Inflammatory Drugs, which act as non-selective cyclooxygenase inhibitors (e.g., Ibuprofen, Diclofenac), are reported to cause different upper and lower gastrointestinal (GI) adverse effects including dyspepsia, heartburn, ulceration, or bleeding, which are the consequence of weakened mucosal protection. Moreover, the free carboxylic group, which is characteristic for most of NSAIDs, is irritating in direct contact with mucosa cells. Those drugs are not ionized in acidic stomach environment, but get dissociated when got into epithelial cells. The effect of ionic trap is responsible for topical mucosal damage. Selective COX-2 inhibitors (COXIBs) exert similar analgesic and anti-inflammatory properties to traditional NSAIDs and, at the same time, show very low gastrotoxicity. Nevertheless, some gastric complications could occur anyway during therapy with COXIBs because COX-2 is also, to some extent, engaged in gastroduodenal mucosa defense. Additionally, chronic use of COXIBs can elevate the risk of serious cardiovascular complications [7,8,9,10,12,13,14,15,16].

Severe adverse effects of both non-selective and selective NSAIDs significantly restrict the usage of these drugs, especially in long-term therapy. Therefore, there is still an urgent prompt for search and develop new, potent, selective, and primarily safe cyclooxygenase inhibitors, which could be deprived of side effects distinctive for known NSAIDs [7,8,14,17,18,19].

**Figure 1:**
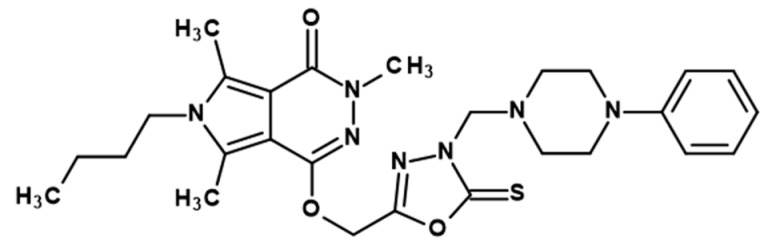
The structure of the most potent new Mannich base-type derivative of pyrrolo[3,4-*d*]pyridazinone.

Our previous study reported the synthesis and biological evaluation of several pyrrolo[3,4-*d*]pyridazinone derivatives with promising anti-inflammatory and anti-oxidant activity. All designed compounds efficiently inhibit cyclooxygenase, have a better affinity to isoenzyme COX-2 and show a superior COX-2/COX-1 selectivity ratio compared to Meloxicam, which was used as the reference drug. According to a molecular docking study, novel Mannich base-type derivatives of pyrrolo[3,4-*d*]pyridazinone take a position in COX’s active site very similar to Meloxicam [20,21]. In relation to performed multiple-criteria decision analysis (MCDA), the most potent compound in formerly carried out study was 6-butyl-3,5,7-trimethyl-1-[[3-[(4-phenylpiperazin-1-yl)methyl]-2-thioxo-1,3,4-oxadiazol-5-yl]methoxy]pyrrolo[3,4-*d*]pyridazin-4-one **1** (Figure 1) [20].

These findings encouraged us to perform further modifications on the investigated scaffold of pyrrolo[3,4-*d*]pyridazin-4-one, based on the structural optimalization of derivative **1** to obtain more effective molecules.

**Figure 2:**
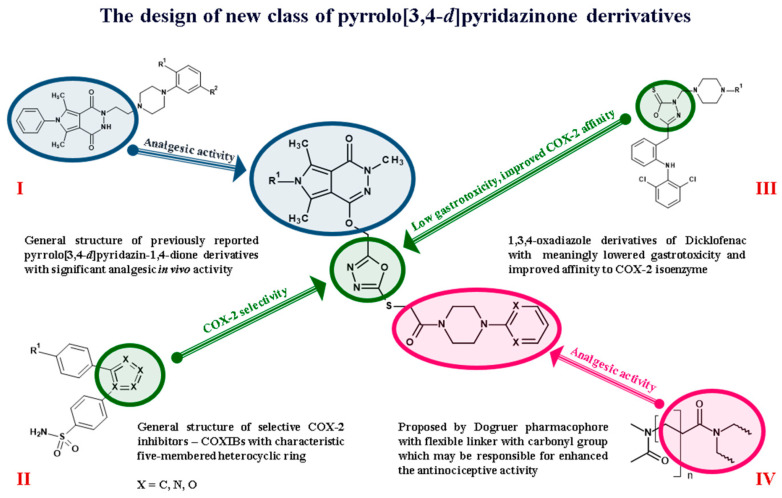
The outline of the concept of the uprising of novel pyrrolo[3,4-*d*]pyridazinone derivatives.

As was mentioned already, derivatives of pyrrolo[3,4-*d*]pyridazinone are proved to have promising analgesic and anti-inflammatory activity (Figure 2, I) [22,23]. We have previously introduced to the biheterocyclic scaffold of pyrrolo[3,4-*d*]pyridazinone, the five-membered moiety of 1,3,4-oxiadiazole-2-thione [20]. This modification was inspired by the fact that in the structure of selective COX-2 inhibitors—COXIBS, characteristic five-membered heterocyclic rings such as isoxazole (Valdecoxib) or pyrrazole (Celecoxib) can be distinguished (Figure 2, II) [18]. Moreover, 1,3,4-oxiadiazole-2-thione can serve as a bioisostere of the carboxylic group [17,24,25,26]. There are plenty of compounds containing the mentioned ring that lack gastrointestinal toxicity while presenting promising anti-inflammatory and analgesic activity. Replacement of irritating free carboxylic group by 1,3,4-oxiadiazole-2-thione moiety in popular NSAIDs, such as Diclofenac or Ibuprofen, allowed to obtain structures with significantly reduced gastrotoxicity and improved COX-2 affinity (Figure 2, III) [27,28]. Finally, the current study’s key modification was the introduction to the new derivatives, the arylpiperazine pharmacophore, through a flexible 2-oxoethyl linker. This structural alteration closely refers to the theory featured by Dogruer (Figure 2, IV) [29,30]. According to this hypothesis, the presence of carbonyl moiety in the alkyl chain could enhance the analgesic and anti-inflammatory activity of new structures.

Taking the above into consideration, we can conclude that new pyrrolo[3,4-*d*]pyridazinone derivatives were designed and synthesized on the base of the idea of the double pharmacophore approach. The 1,3,4-oxadiazole-2-thione moiety is one of the most valid in contemporary medicinal chemistry. It is present in a great variety of compounds that exert diverse biological activities, including cyclooxygenase inhibitors, as well [18,19,31,32]. On the other hand, arylpiperazine pharmacophore occurs in many potent antinociceptive and anti-inflammatory agents [20,22,23,27,28,29,30]. Herein, in the structure of title compounds, both 1,3,4-oxadiazole, arylpiperazine pharmacophore and flexible 2-oxoethyl linker, proposed by Dogruer [29,30], could be recognized (Figure 2). Hopefully, such a synthetic approach will result in the obtainment of potent bioactive molecules.

Currently, we present the synthesis, comprehensive in vitro and in silico evaluation of pyrrolo[3,4-*d*]pyridazinone derivatives designed as a potential novel class of analgesic anti-inflammatory agents inhibiting cyclooxygenase. This study’s aim is to determine the impact of the mentioned elongated 2-oxyethyl linker on the anti-inflammatory and anti-oxidant activity of new pyrrolo[3,4-*d*]pyridazinone derived compounds. All performed complex investigations focus on possibly the most precise explanation of the probable mechanism of action of new molecules. Moreover, we determine their cytotoxicity and model of interaction with blood proteins.

## 2. Results and Discussion

### 2.1. Chemistry

**Scheme 1:**
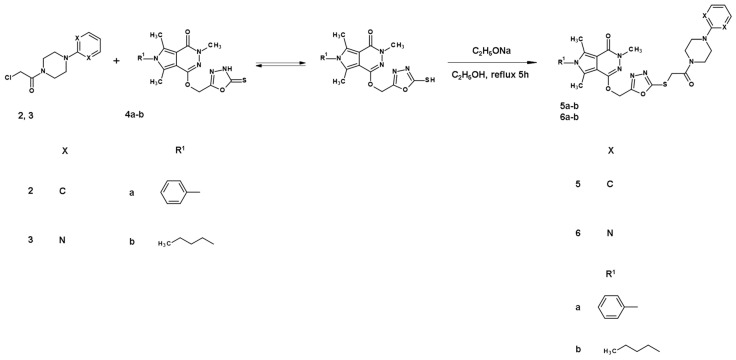
The synthesis and structures of final pyrrolo[3,4-*d*]pyridazinone derivatives **5a,b**–**6a,b**.

The objective of this study was the design and synthesis of novel pyrrolo[3,4-*d*]pyridazinone derivatives. The structures of final compounds **5a,b**–**6a**,**b** (Scheme 1) were based on already published in the literature the structure–activity relationships of different analgesic and anti-inflammatory agents (Figure 2).

The first step of the planned synthesis assumed the obtainment of appropriate arylpiperazine pharmacophores. The alkylation of 1-phenylpiperazine or 1-(2-pyrimidyl)-piperazine with chloroacetyl chloride led to the formation of 1-(2-chloro-1-oxoethyl)-4-phenylpiperazine **2** and 1-(2-chloro-1-oxoethyl)-4-(2-pyrimidyl)-piperazine **3**, respectively. This stage was performed according to previously reported protocols [33]. The synthesis of 3,5,7-trimethyl-6-phenyl-1-[(2-thioxo-3*H*-1,3,4-oxadiazol-5-yl)methoxy]pyrrolo[3,4-*d*]pyridazin-4-one **4a** and 6-butyl-3,5,7-trimethyl-1-[(2-thioxo-3*H*-1,3,4-oxadiazol-5-yl)methoxy]pyrrolo[3,4-*d*]pyridazin-4-one **4b** was carried out also in accordance to our procedure which has been published already [20]. Scheme 1 presents the synthesis of compounds which have not been described in the literature yet. All spectroscopic and analytical properties of all afresh received derivatives were in good agreement with their predicted structures and are summarized in the experimental section and in Supplementary Data.

The title compounds **5a**,**b**–**6a**,**b** were synthesized with satisfactory yield in a convenient way through the alkylation of key pyrrolo[3,4-*d*]pyridazinone analogues **4a** and **4b** with arylpiperazine derivatives **2** or **3**, respectively. The final reactions were carried out in ethanol in the presence of sodium ethoxide, which played the role of hydrogen chloride binding factor (Scheme 1). The thin-layer chromatography technique was used to monitor the progress of the reaction. The precipitated crude products were filtered off, washed with ethanol, and purified by crystallization from proper solvent.

Due to the occurrence of tautomerism in the 1,3,4-oxadiazole-2-thione ring, the alkylation of appropriate analogue of pyrrolo[3,4-*d*]pyridazinone **4a**,**b** with Suitable 2-chloro-1-oxoethylarylpiperazine derivative **2**, **3** may result in obtainment of a mixture of isomeric *N*- and *S*--forms. In the current study, the applied reagents and reaction conditions allowed the formation of, exclusively, *S*-isomers **5a**,**b**–**6a**,**b** (Scheme 1). Such a conclusion was drawn based on the analysis of the NMR spectra.

When considering the ^13^C NMR spectra of compounds **4a**,**b**, the significant peak near 177.89–178.90 ppm, which is typical for carbon atom which forms C=S bond, can be distinguished. The lack of this characteristic signal in the ^13^C NMR spectra of the final structures **5a**,**b**–**6a**,**b** (experimental section and Supplementary Data) indicates that the title compounds were formed via *S*-alkylation of 1,3,4-oxadiazole-2-thiole derivatives of pyrrolo[3,4-*d*]pyridazinone **4a**,**b** (Scheme 1). This is also confirmed by the presence of characteristic peak near 165.36-165.42 ppm in ^13^C NMR spectra of **5a**,**b**–**6a**,**b** which can be assigned to carbon C2 in 1,3,4-oxadiazole, which binds sulfur via single bond.

Continuing the analysis of the ^13^C NMR spectra of final molecules **5a**,**b**–**6a**,**b** we can observe peaks which are the signals of carbon atoms present in 2-oxoethyl linker. A peak near 164.59–164.81 ppm is typical for carbonyl (C=O), whereas a signal in the area of 32.32 ppm is assigned to carbon atom 1C (-CH_2_-). Furthermore, the presence of an additional two-proton singlet, which appears, depending on the considering derivative, in the range of 4.33-4.42 ppm in ^1^H NMR spectra of **5a**,**b**–**6a**,**b** could be equated with methylene group present in 2-oxoethyl linker.

In our former study, the compounds bearing phenylpiperazine moiety (Figure 1) appeared to be the most potent in performed experiments [20]. That is why we have decided to introduce the same pharmacophore to one series of novel derivatives. In the second series, a pyrimidine ring is present to evaluate the impact of this terminal aromatic six-membered ring on new compound activity. Moreover, the structure of new agents will allow us to estimate the influence of elongated 2-oxoethyl linker and the substitution of the sulfur atom in 1,3,4-oxadiazole-2-thiol ring on the pharmacological activity of new pyrrolo[3,4-*d*]pyridazinone derivatives

The crude intermediate and final products were purified by crystallization from the suitable solvent. Structures of all newly synthesized derivatives were established and confirmed by spectroscopic techniques including ^1^H NMR, ^13^C NMR, MS, FT-IR, elemental analysis and based on their physicochemical properties.

### 2.2. Cyclooxygenase (COX-1, COX-2) Inhibition Studies

#### 2.2.1. In Vitro Cyclooxygenase Inhibition Assay

The tested compounds’ ability to inhibit the activity of COX-1 and COX-2 was assessed by Cayman’s COX Colorimetric Inhibitor Screening Assay (cat. no. 701050). Each sample was prepared in triplicate at a concentration of 100 µM. The study started with 2 min incubation at RT, and then peroxidase activity was measured with Varioskan LUX microplate reader (Thermo Scientific) at a wavelength of 590 nm. The outcome is shown as the IC_50_ values, i.e., the concentrations at which a 50% inhibition of enzyme activity appeared for both COX-1 and COX-2. Meloxicam, which shows better affinity to COX-2 than to COX-1, therefore is used by patients with GI complications, was used as a reference compound. The results for both enzymes are presented in the table below. The study showed that the tested structures inhibit the activity of COX-2 enzymes and have no impact on the COX-1 isoform (Table 1). The most active compound appeared to be **6a**, in which 50% inhibition of COX-2 enzyme activity occurred at the lowest concentration. Taking these results into account, it is worth emphasizing that both isoforms of cyclooxygenase have slight but important structural differences in their active sites. The isoleucine (Ile) residue in position 523 in COX-1 isoform is replaced by valine (Val) when considering isoform COX-2. Furthermore, in the case of COX-1, in position 434 occurs isoleucine (Ile) and in position 513 histidine (His), while in COX-2 valine (Val) and arginine (Arg), respectively. Due to these differences, the size of the COX-2 pocket is bigger than that of COX-1, which allows selective binding of larger molecules [1,2,3,4,5]. In the case of the COX-2 enzyme, investigated compounds displayed lower activity than the reference.

When comparing the COX inhibitory activity of new compounds **5a,b–6a,b** with previously reported Mannich base-type derivatives of pyrrolo[3,4-*d*]pyridazinone [20] we can point out some important differences between these two series. Most of formerly studied compounds had the affinity towards both isoenzymes of cyclooxygenase, with superior activity on COX-2, while molecules described in this manuscript act as selective COX-2 inhibitors. Basing on these findings, and with reference to investigations performed earlier by Malinka et al. [22,23] we can try to speculate about some structure–activity relationships in the group of analgesic and anti-inflammatory agents based on pyrrolo[3,4-*d*]pyridazinone core.

Malinka et al. reported the synthesis and extensive biological evaluation of new derivatives of pyrrolo[3,4-*d*]pyridazinone with promising antinociceptive activity. In the structure of described compounds, the characteristic arylpiperazine/piperidine pharmacophore can be recognized. It was attached directly to the pyridazinone core via different flexible linkers such as ethyl, propyl or 2-hydroxypropyl. Despite good pharmacological activity, the exact mechanism of action of these compounds has not been solved yet [22,23].

Inspired by those results we decided to continue this work and modify the pyrrolo[3,4-*d*]pyridazinone scaffold by incorporation of the five-membered 1,3,4-oxadiazole-2-thione moiety with the hope of receiving potent cyclooxygenase inhibitors with good affinity towards COX-2. As was mentioned before, we have achieved the intended goal. The 1,3,4-oxadiazole ring is supposed to enhance COX inhibitory activity of our compounds [20]. When considering derivatives **5a,b–6a,b** we can infer that introduction of arylpiperazine pharmacophore via flexible 2-oxoethyl linker to the pyrrolo[3,4-*d*]pyridazinone-based 1,3,4-oxadiazole molecules resulted in their COX-2 selectivity. The fact that in the current study we report molecules received by *S*, not *N* alkylation of 1,3,4-oxadiazole-2-thiol, could also be significant. Nevertheless, certainly further extensive studies and subsequent structural modifications of future derivatives are needed, because the increase of selectivity led to a decrease of activity.

#### 2.2.2. Cyclooxygenase Molecular Docking Study

To estimate the possible binding interactions of synthesized compounds inside the active site of cyclooxygenase, a molecular docking study was performed. AutoDock software was used. The scoring function binding free energy (ΔG°) for interaction with COX-1 for all tested compounds showed a positive value. This result is in very good correlation with a biological evaluation, which indicated no activity towards the COX-1. The docking results for interactions with COX-2 are assumed in Table 2. The ΔG° is negative for all compounds. The lowest value was found for **6b**. For all compounds, the sum of van der Waals energy, hydrogen bonding energy, and desolvation free energy (ΔE_2_) is more negative than electrostatic energy (ΔE_3_). It indicates that the main interactions are van der Waals and hydrogen bonding interactions. For **6a** and **5b**, inhibit constants obtained from molecular docking showed lower values than for **6b** and **5a**.

**Figure 3:**
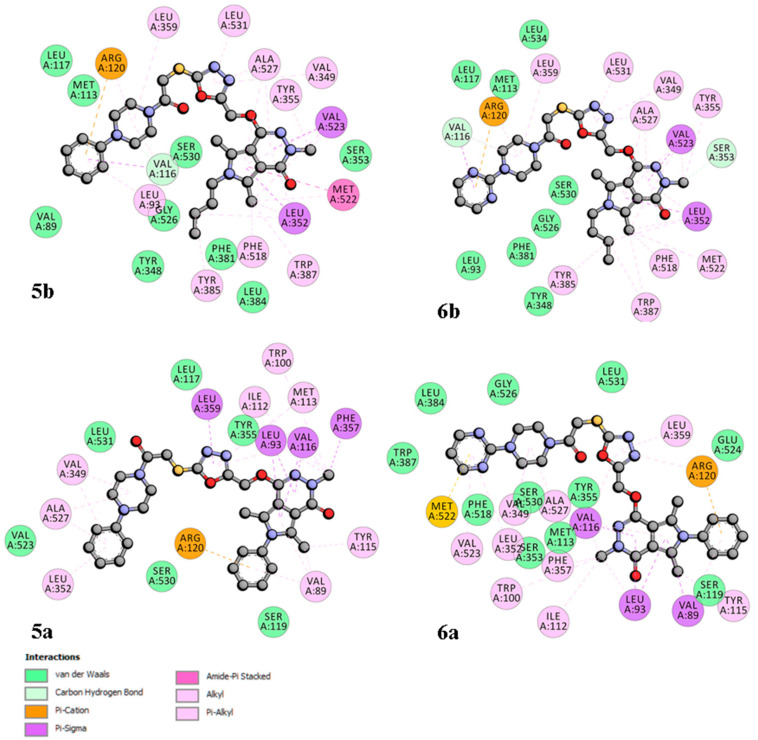
2D interaction plot of **5a**, **5b**, **6a**, **6b** with COX-2.

**Figure 4:**
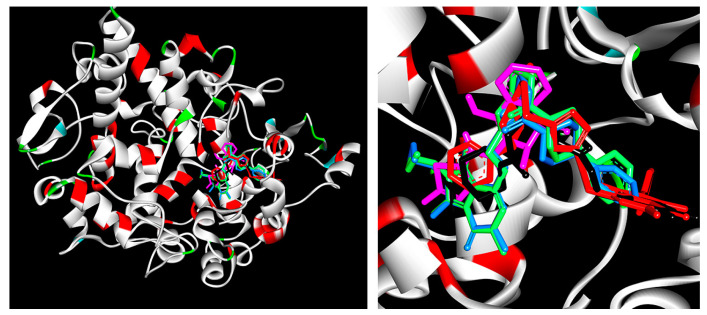
Docking poses of studied compounds **5a**,**b**–**6a**,**b** and Meloxicam (pink) inside COX-2.

Inside the COX-2 active site, no conventional hydrogen bond interaction was found. Hydrophobic interactions play an important role. The details are presented in Figure 3. The COX ligand binding site has four specific subdomains: A, B, C, and D. Subdomain A represents the mode of binding of flurbiprofen; subdomain B represents the mode of binding of Meloxicam and Piroxicam; subdomain C represents an entrance region of the enzyme binding domain, and subdomain D represents the position of the residue in position 523 [34]. The size of the COX-2 pocket is bigger than that of COX-1., It allows the selective binding of larger molecules. The position of studied compounds was found very similar to Meloxicam (Figure 4).

### 2.3. Evaluation of Viability

**Figure 5:**
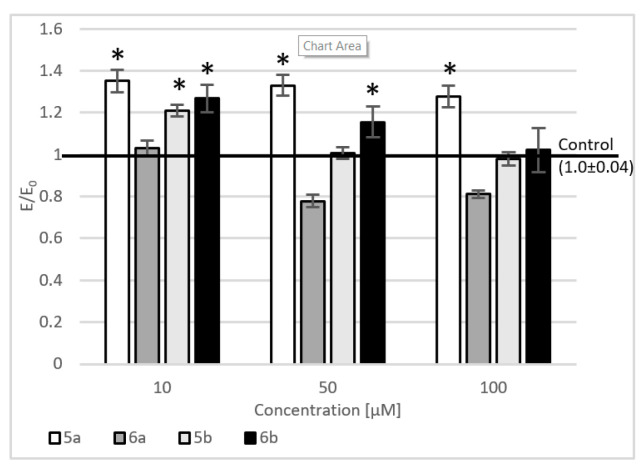
Effect of tested compounds on Normal Human Dermal Fibroblasts (NHDF) cells; metabolic activity measured in MTT assay; Control—cell culture incubated without tested substances; * *p* < 0.05—significant difference compared to control.

Dependence between viability and concentration was observed for all investigated compounds (Figure 5). The study showed that the metabolic activity of **5b** and **5a** decreases as the concentration increases, whereas **6a** toxicity at 50 µM concentration is higher than at 100 µM, but it is within experimental error. None of the cases showed a decrease of viability under 30%, indicating a lack of tested compound cytotoxic potential. Furthermore, **5a** and **5b** at 10 µM concentration presumably improve the proliferation of Normal Human Dermal Fibroblasts (NHDF) cells. Merely **6a** at 50-100 µM concentration range revealed mild cytotoxicity of a dozen or so percent.

### 2.4. Level of Intracellular Reactive Oxygen Species, Nitric Oxide, and DNA Damage

**Scheme 2:**
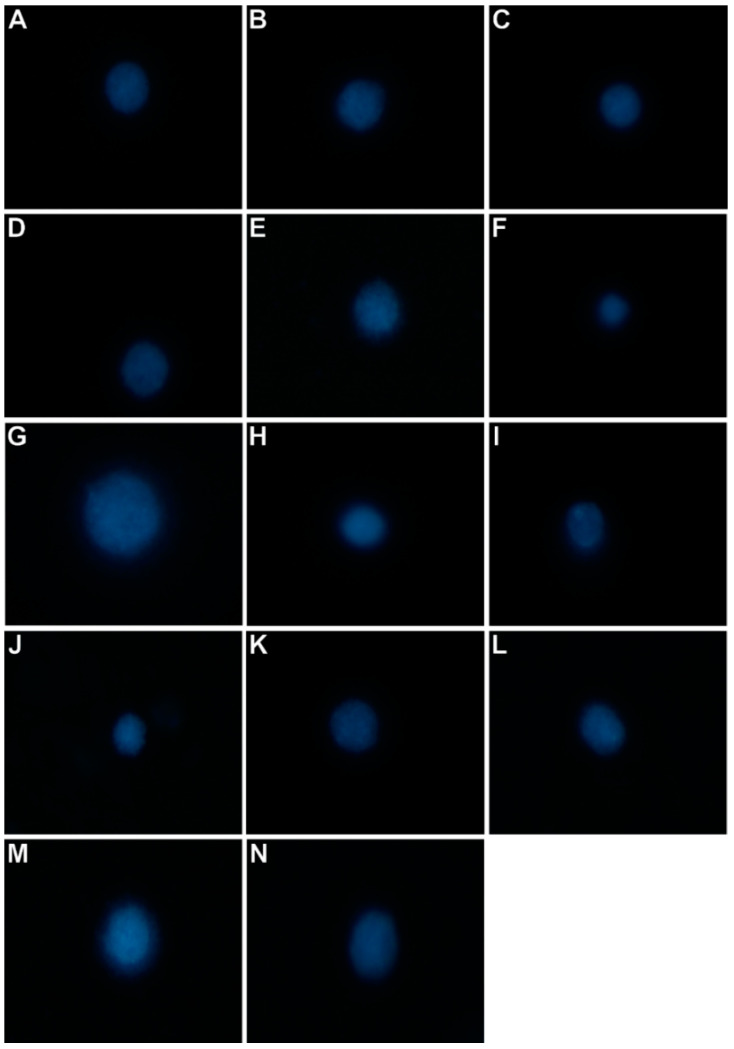
Micrographs (60×) of cells showing chromatin relaxation: (**A**) compound **5a** at 10 µM; (**B**) compound **5a** at 50 µM; (**C**) compound **5a** at 100 µM; (**D**) compound **5b** at 10 µM; (**E**) compound **5b** at 50 µM; (**F**) compound **5b** at 100 µM; (**G**) compound **6a** at 10 µM; (**H**) compound **6a** at 50 µM; (**I**) compound **6a** at 100 µM; (**J**) compound **6b** at 10 µM; (**K**) compound **6b** at 50 µM; (**L**) compound **6b** at 100 µM; (**N**) cell incubated in the complete medium; (**M**) cell incubated with 100 µM H_2_O_2_ for 1 h.

For all tested compounds, a relationship between the level of free oxygen radicals, nitric oxide or the number of DNA strand breaks and concentration was observed (Table 3). The results showed that the level of free oxygen radicals after using all tested compounds is the lowest at a concentration of 10 µM. After using compounds **5a**, **5b**, **6b**, a decrease in the level of free oxygen radicals below the positive control was noticed (cell culture treated with only 100 µM H_2_O_2_) in the entire range of tested concentrations. Compound **6a** at concentrations of 50 and 100 µM did not reduce the level of free oxygen radicals. Regarding the nitric oxide (NO) level, a reduction in NO levels was observed after incubating the culture with the tested compounds compared to the positive control after incubation at a concentration of 10 and 50 µM for **6b** and **5a**, 10 µM for **5b** and in the entire concentration range for **6a**. At the same time, a reduction in the amount of DNA strand damage after incubation with the investigated compounds was demonstrated for all tested concentrations for **6b** and **5a**, at 10 and 50 µM for compound **5b** and the lowest tested concentration for compound **6a** (Scheme 2).

Correlation coefficients between the DNA damage assay results and the level of free oxygen radicals or nitric oxide were calculated (Table 4). In all cases, strong positive correlations were observed between the levels of reactive oxygen species (ROS) and NO as well as DNA strand breakage. These results may suggest a strong reparative effect (scavenging of free radicals and repair of DNA strand breaks) of the tested compounds in a state of increased exogenous stress (incubation of cell cultures with H_2_O_2_), possibly by inhibiting COX-2.

### 2.5. Fluorescence Quenching and Binding Constants

**Figure 6:**
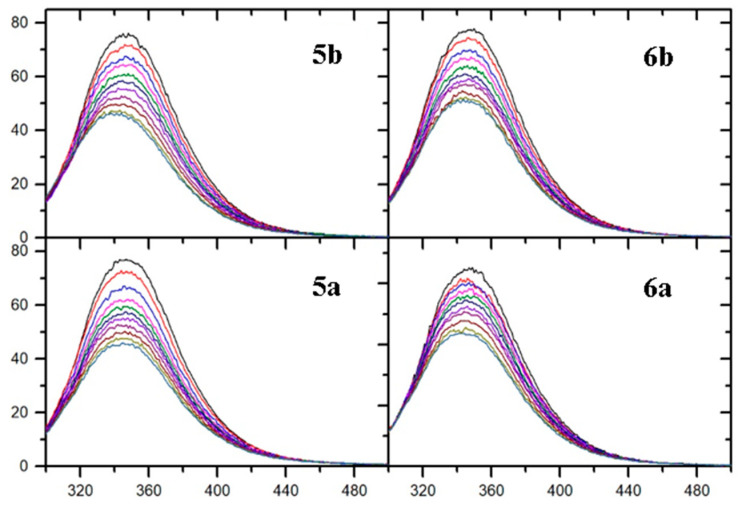
Fluorescence spectra of Bovine Serum Albumin solution in the presence of **5a**, **5b** and **6a**, **6b** (T-294 K, λex = 280 nm). The concentration of **5a**, **5b** and **6a**, **6b** was: 0, 0.2, 0.4, 0.6, 0.8, 1.0, 1.2, 1.4, 1.6, 1.8, 2.0 µM.

To investigate the binding properties of **5a**, **6a**, **5b**, and **6b** to Bovine Serum Albumin, the fluorescence spectra were recorded in the range of 300–500 nm upon excitation at 280 nm (both Trp and Tyr residues are excited) and concentration range 0.0–2.0 µM (Figure 6). When different amounts of tested compounds were titrated, BSA’s fluorescence intensity decreased, suggesting that all compounds could interact with BSA and quench its intrinsic fluorescence. The presence of **5a**, **6a**, **5b**, and **6b** also caused a blue shift in the maximum emission wavelength of protein. It indicates that BSA’s conformation and the amino acid residues are located in a more hydrophobic environment and are less exposed to the solvent [35]. Fluorescence quenching and shift of λ_max_ identify interaction with BSA and suggest the formation of complexes (static quenching). However, it can also be the result of the collisional encounters (dynamic quenching). To confirm the quenching mechanism and complex formation, the fluorescence data were further analyzed by the Stern–Volmer equation and dependence on temperature [36].

The fluorescence data were analyzed at three different temperatures, 294, 301, 308 K, using Stern–Volmer Equation [37] (Equation (S1) in the Supplementary Data) after correction due to the infer filter effect (Equation (S2) in the Supplementary Data). The Stern–Volmer (K_SV_) constant was determined by the linear fitting. The calculated results are collected in Table 5. The K_SV_ values decreasing with increasing temperature, and the quenching rate constant (k_q_) values are much greater than the value of the maximum scatter collision quenching constant (2 × 10^10^ dm^3^·mol^−1^·s^−1^ [38]). It indicated that the probable quenching mechanism is static rather than dynamic, and it suggested the formation of a ground-state complex.

**Figure 7:**
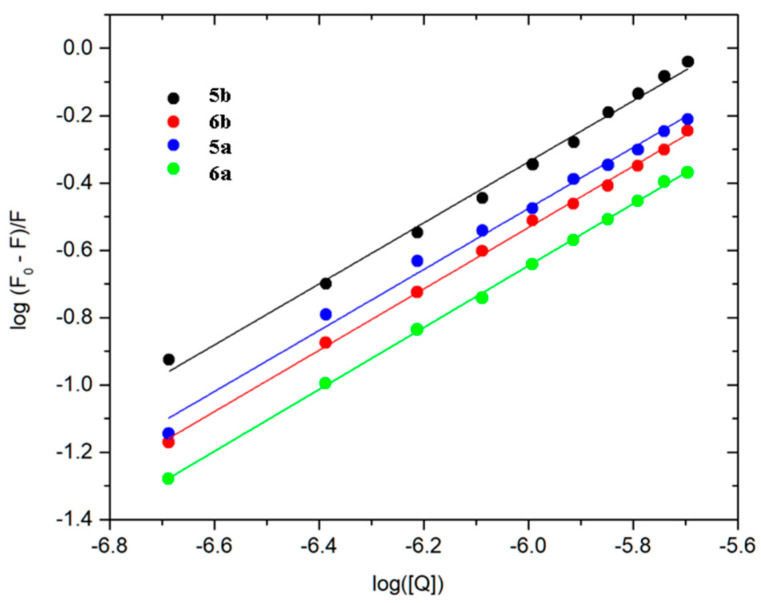
Double logarithm regression plots for quenching of BSA by **5a**, **5b**, **6a**, and **6b.**

The binding constants and the number of binding sites were calculated using a double logarithm regression curve (Equation (S3) in the Supplementary Data). As is shown in Figure 7 there is a good linear fit for all studied compounds. The calculated results are listed in Table 5. The results showed that the binding constants indicate values about 10^5^ dm^3^·mol^−1^ at 294 K and decreasing with increasing temperature. The differences between **5a**, **6a**, **5b**, and **6b** compounds are not great. The number of the binding site is close to 1, shows one-to-one interaction. The interaction of 14 anti-inflammatory drugs with human serum albumin was investigated by F. Mohammadnia [39]. The binding constants were found with the range 10^2^ dm^3^·mol^−1^ (acetaminophen) to 1.88 × 10^7^ dm^3^·mol^−1^ for Meloxicam. Therefore, K_b_ values of studied compounds show that the interactions with BSA are moderate. Similar values were obtained for many compounds with biological activity [20,36,40,41,42,43,44].

### 2.6. Site Markers Studies and Molecular Docking

BSA, as well as human serum albumin (HSA), is known to possess two binding sites, which are situated in subdomains IIA and IIIA [45]. To confirm the binding sites on BSA involved in **5a**, **6a**, **5b**, and **6b** binding, phenylbutazone (PHB) and ibuprofen (IBP) were used as site probes [46]. Binding constants were analyzed using Equation (S3) (in the Supplementary Data). Calculated values were found as 5.50 × 10^3^ (**5b**), 4.79 × 10^3^ (**6b**), 4.17 × 10^3^ (**5a**), 3.24 × 10^3^ (**6a**). The results show that binding constant Kb of all tested compounds with BSA in the presence of IBP considerably decline compared to compound BSA only (Table 5.). In the presence of PHB, Kb value showed smaller difference compared to no marker: 4.27 × 10^4^ (**5b**), 3.31 × 10^4^ (**6b**), 4.07 × 10^4^ (**5a**), 2.51 × 10^4^ (**6a**). It can be suggested that **5a**, **5b**, **6a**, **6b** mainly bind to the subdomain IIIA of BSA.

**Figure 8:**
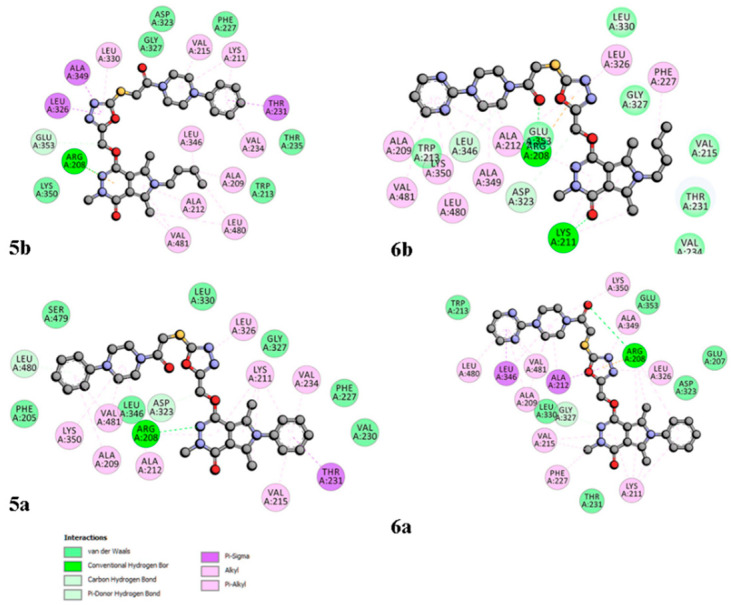
2D interaction plot of **5a**, **5b**, **6a**, **6b** with BSA in the binding site II (subdomain IIIA).

The binding interactions between studied compounds and BSA were simulated by the molecular docking method. The results were presented in Table 6. The results demonstrated that the binding free energy within the hydrophobic cavity in site II (subdomain IIIA) of BSA was more negative than that within the hydrophobic cavity in site I (subdomain IIA). It indicates that site II is favorable. Such outcome is consistent with the results observed in the site marker fluorescence studies. The sum of van der Waals energy, hydrogen bonding energy, and desolvation free energy (ΔE_2_) is more negative than electrostatic energy (ΔE_3_). Hence, it can indicate that the main interactions are van der Waals and hydrogen bonding interactions. In binding site IIIA studied compounds are surrounded by various kinds of residues (Figure 8). Hydrogen bonds with Arg208 (**5a**, **5b**, **6a**, **6b**) and Lys211 (**6b**) are formed. The π-sigma and other hydrophobic interactions are observed. The details are presented in Figure 8.

The interaction forces between a small molecule and protein include hydrogen bond, van der Waals force, electrostatic and hydrophobic interactions, etc. [47]. The thermodynamic parameters enthalpy change (ΔH°), the entropic change (ΔS°) and free energy change (ΔG°) were calculated from Equations (S4) and (S5) in the Supplementary Data. The results are listed in Table 5. We can conclude that the binding interaction between tested compounds and BSA were spontaneous due to the negative ΔG°. Both the ΔH° and ΔS° negative values indicate that the main interaction force in the binding process was van der Waals forces and/or hydrogen bonding interaction.

### 2.7. Circular Dichroism Spectra

**Figure 9:**
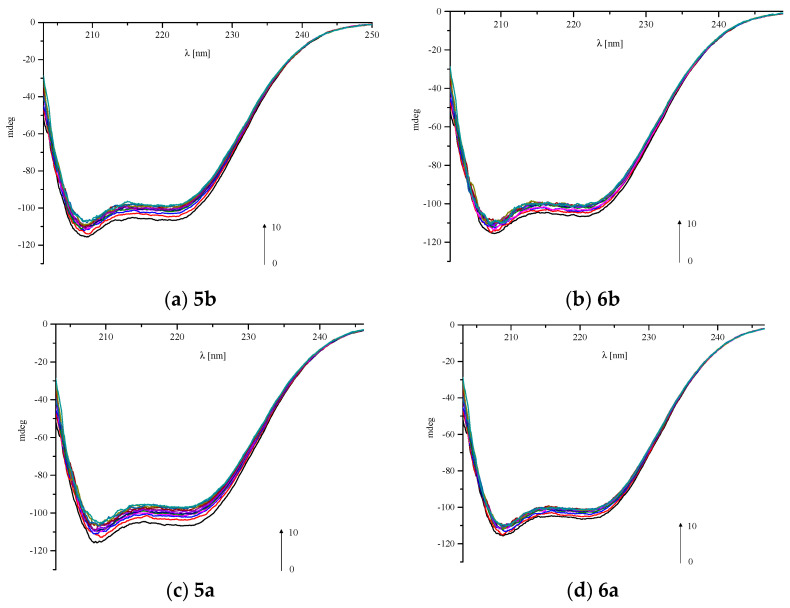
CD spectra of BSA in the absence and presence of all analyzed compounds. BSA to analyzed compounds molar ratios was changing from 1:0 to 1:10.

Circular dichroism (CD) spectroscopy is a useful method to determine the secondary structure changes in the conformation of proteins. It can check the interaction between the protein molecule and new pharmaceutical compounds [48]. In this study, we had monitored the changes in BSA structure when four analyzed compounds: **5a**, **5b**, **6a**, and 6b were absent or present in solutions. In all CD spectra, we observed two negative bands at near 208 nm and 222 nm, typical for the α-helical structure of the protein (Figure 9). Any changes in this region suggest conformational changes in protein molecules [49].

Figure 9 shows that we observed the reduction of ellipticity values at 208 nm and 222 nm in the presence of all analyzed compounds. Any shift of the peaks was not observed. To measure the observed change size, we calculated the α-helix(%) values after adding every portion of each analyzed compound (Table 7).

The content of α-helix can be calculated using Equations (1) and (2) [50]:

(1) $$\alpha -helix\left(\%\right)=\frac{-MRE_{208}-4000}{33,000-4000}100\%$$

where *MRE*_208_ is the *MRE* value observed at 208 nm, 4000 and 33,000 is the *MRE* value of the β-form and random coil conformation cross at 208 nm value of pure *α*-helix at 208 nm, respectively.

(2) $$MRE=\frac{ObservedCD\left[mdeg\right]}{10Cnl}$$

*C* is the molar concentration of BSA, n is the number of amino acid residues 583 for BSA, and l is the path length in cm.

**Figure 10:**
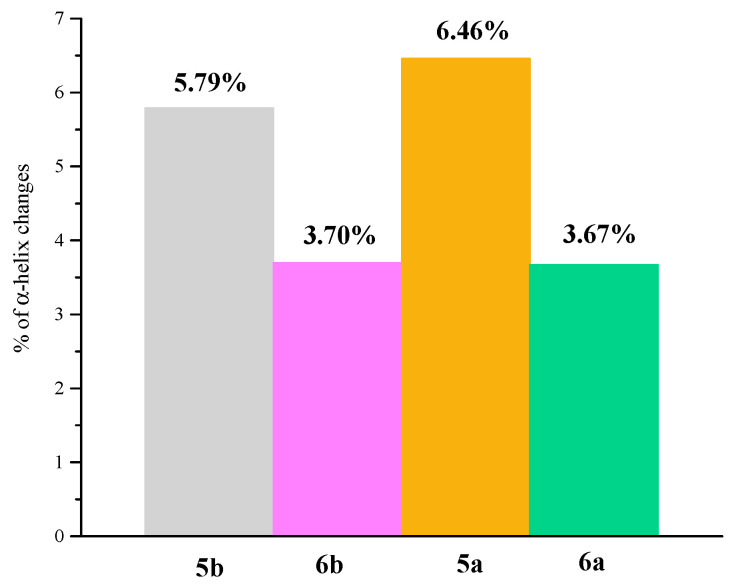
Changes in α-helix(%) of BSA after adding 10 portions of the compound—the comparison for **5a**, **5b**, **6a**, and **6b**.

The results collected in Figure 9 and Figure 10 and Table 7 show that greater changes of α-helix(%) are observed in the case of **5b** and **5a**. The α-helical content of BSA decreased from 53.55% to 47.76% and from 54.05% to 47.59%, respectively, when BSA to analyzed compounds molar ratio was increasing from 1:0 to 1:10. The smallest changes were observed after adding **6a** to BSA (from 53.78% to 50.11%, Table 7, Figure 9). Above 1:6 BSA to **6a** molar ratio, there was no longer any changes. The greatest changes in the CD spectra are observed in case of 1:0.5 and 1:1 molar ratios. This result is consistent with the fluorescence measurements and molecular docking studies, where it was proved that analyzed compounds can form complexes with BSA in 1:1 ratios. Further changes observed after adding next portions of analyzed ligands may be caused by the interaction of hydrophobic sites of the protein with hydrophobic groups in compounds. Changes in the α-helical content of BSA for all four compounds are compared in Figure 10.

## 3. Materials and Methods

### 3.1. Chemistry

#### 3.1.1. Instrumentation and Chemicals

All chemicals, reagents, and solvents used in the current study were purchased from commercial suppliers (Chemat, Gdańsk, Poland; Archem, Łany, Poland; Alchem, Wrocław, Poland) and used without further purification. Dry solvents were obtained according to the standard procedures. Progress of the reaction was monitored by thin-layer chromatography (TLC) technique on silica-gel-60-F254-coated TLC plates (Fluka Chemie GmbH) and visualized by UV light at 254 or 366 nm. Chromatographic separations and purifications were performed on a silica-gel [Kieselgel 60 (70–230 mesh), Merck] column (CC). The melting points of received products were determined by an open capillary method on Electrothermal Mel-Temp 1101D apparatus (Cole-Parmer, Vernon Hills, IL, USA) and were uncorrected. The ^1^H NMR (300 MHz) and ^13^C NMR (75 MHz) spectra were recorded on a Bruker 300 MHz NMR spectrometer (Bruker Analytische Messtechnik GmbH, Rheinstetten, Germany) in CDCl_3_ or DMSO-*d_6_* using tetramethylsilane (TMS) as an internal reference. Chemical shifts (δ) are reported in ppm. Spectra were recorded and read using TopSpin 3.6.2. Bruker Daltonik, GmbH, Bremen, Germany) The infrared (IR) spectra were determined on a Nicolet iS50 FT-IR Spectrometer (Thermo Fisher Scientific, Waltham, MA, USA). Samples were applied as solids, and frequencies are reported in cm^−1^. Spectra were read using OMNIC Spectra 2.0 Thermo Fisher Scientific, Waltham, MA, USA) Mass spectra were recorded using a Bruker Daltonics Compact ESI-mass spectrometer (Bruker Daltonik, GmbH, Bremen, Germany). The instrument was operated in positive ion mode. Analyzed compounds were dissolved in a mixture of chloroform and methanol. Elemental analyses for carbon, nitrogen and hydrogen were run on a Carlo Erba NA-1500 analyzer (Thermo Fisher Scientific, Waltham, MA, USA), and obtained results were within ± 0.4% of the theoretical values calculated for corresponding formulas.

#### 3.1.2. Chemical Synthesis

The synthesis protocols and experimental data for derivatives **4a**,**b** and all intermediates were previously reported[20,33].

General procedure for preparation of title derivatives of pyrrolo[3,4-d]pyridazinone 5a,b–6a,b.

The appropriate derivative of pyrrolo[3,4-*d*]pyridazinone 4a or 4b (0.001 mol) was suspended in 30mL of anhydrous ethanol in a round bottom flask. Then, the 1mL of 1M sodium exthoxide (0.001 mol) and appropriate arylpiperazine derivative 2 or 3 was added, and the mixture was refluxed for 6h. The reaction progress was monitored by TLC. The mixture was left overnight. After cooling, the precipitate was formed. The solid was filtered off, washed thoroughly with ethanol, and afterwards purified by crystallization from ethanol.

**5a:** 3,5,7-trimethyl-1-[[2-[2-oxo-2-(4-phenylpiperazin-1-yl)ethyl]sulfanyl-1,3,4-oxadiazol-5-yl]methoxy]-6-phenyl-pyrrolo[3,4-*d*]pyridazin-4-one

White solid, Yield: 76,91%; m.p.: 186–188 °C

FT-IR (selected lines, γ_max_, cm^−1^): 3067 (C-H arom.), 2916 (C-H aliph.), 1646 (C=N)

^1^H NMR (300 MHz, CDCl_3_): δ= 2.27 (s, 3H, 7-CH_3_), 2.45 (s, 3H, 5-CH_3_), 3.19–3.21 (m, 2H, CH_2_-piperazine), 3.22–3.26 (m, 2H, CH_2_-piperazine), 3.59 (s, 3H, 3-CH_3_), 3.76 (m, 2H, CH_2_-piperazine), 3.84 (m, 2H, CH_2_-piperazine), 4.41(s, 2H, S-CH_2_), 5.49 (s, 2H, O-CH_2_), 6.95-6.98 (m, 3H, ArH), 7.19–7.22 (m, 2H, ArH), 7.31–7.33 (m, 2H, ArH), 7.54–7.57 (m, 3H, ArH)

^13^C NMR (75 MHz, CDCl_3_): δ= 11.41, 11.84, 36.96, 37.10, 42.25, 46.04, 49.45, 49.82, 57.01, 108.42, 112.07, 116.94, 124.24, 127.79, 129.34, 129.73, 130.74, 136.68, 148.06, 159.32, 163.88, 164.60, 165.43

HR-MS (m/z): calcd. for C_30_H_31_N_7_O_4_S [L+H]^+^: 586.2243; found: 586.2231; Anal. calcd. (%) for C_30_H_31_N_7_O_4_S: C:61.52, H:5.34, N:16.74, found: C:61.81, H:5.19, N:16.50,

**5b:** 6-butyl-3,5,7-trimethyl-1-[[2-[2-oxo-2-(4-phenylpiperazin-1-yl)ethyl]sulfanyl-1,3,4-oxadiazol-5-yl]methoxy]pyrrolo[3,4-*d*]pyridazin-4-one

Whitish solid, Yield: 69,44%; m.p.: 141–143 °C

FT-IR (selected lines, γ_max_, cm^−1^): 2964, 2925, 2871 (C-H aliph.), 1645 (C=N)

^1^H NMR (300 MHz, CDCl_3_): δ= 0.87-0.92 (m,3H, -CH_2_-CH_2_ CH_2_-CH_3_), 1.27–1.39 (m, 2H, -CH_2_-CH_2_ CH_2_-CH_3_), 1.57 (m, 2H, -CH_2_-CH_2_-CH_2_-CH_3_), 2.40–2.42 (s, 3H, 7-CH_3_), 2.60–2.61 (s, 3H, 5-CH_3_), 3.13 (m, 2H, CH_2_-piperazine), 3.19 (m, 2H, CH_2_-piperazine), 3.46–3.47 (s, 3H, 3-CH_3_), 3.70 (m, 2H,-CH_2_-CH_2_-CH_2_-CH_3_), 3.77–3.83 (m, 4H, CH_2_-piperazine), 4.33 (s, 2H, S-CH_2_), 5.36–5.37 (s, 2H, O-CH_2_), 6.88-6.90 (m, 3H, ArH), 7.19-7.23 (m, 2H, ArH)

^13^C NMR (75 MHz, CDCl_3_): δ= 10.65, 11.19, 13.69, 20.04, 32.32, 36.90, 37.02, 42.21, 43.94, 46.02, 49.53, 49.88, 56.93, 108.15, 111.85, 122.59, 129.15, 129.36, 147.95, 159.24, 163.95, 164.61, 165.38

HR-MS (m/z): calcd. for C_28_H_35_N_7_O_4_S [L+H]^+^: 566.2549; found: 566.2544; Anal. calcd. (%) for C_28_H_35_N_7_O_4_S: C:59.45, H:6.24, N:17.33, found: C:59.67, H:6.28, N:17.04,

**6a:** 3,5,7-trimethyl-1-[[2-[2-oxo-2-(4-pyrimidin-2-ylpiperazin-1-yl)ethyl]sulfanyl-1,3,4-oxadiazol-5-yl]methoxy]-6-phenyl-pyrrolo[3,4-*d*]pyridazin-4-one

White solid, Yield: 85,27 %; m.p.: 237–239 °C

FT-IR (selected lines, γ_max_, cm^−1^): 3079 (C-H arom.) 2916 (C-H aliph.), 1645 (C=N)

^1^H NMR (300 MHz, CDCl_3_): δ= 2.25 (s, 3H, 7-CH_3_), 2.43 (s, 3H, 5-CH_3_), 3.58 (s, 3H, 3-CH_3_), 3.64–3.66 (m,2H, CH_2_-piperazine), 3.73–3.75 (m, 2H, CH_2_-piperazine), 3.84–3.86 (m, 2H, CH_2_-piperazine), 3.91–3.92 (m, 2H, CH_2_-piperazine), 4.41 (s, 2H, S-CH_2_), 5.47 (s, 2H, O-CH_2_), 6.54-6.58 (m, 1H, ArH), 7.18–7.20 (m, 2H, ArH), 7.53–7.55 (m, 3H, ArH), 8.32–8.34 (m, 2H, ArH)

^13^C NMR (75 MHz, CDCl_3_): δ= 11.40, 11.84, 37.09, 37.13, 42.20, 43.33, 43.59, 45.94, 57.01, 108.42, 110.73, 112.07, 124.24, 127.79, 129.34, 129.73, 130.72, 136.68, 148.05, 157.82, 159.31, 161.40, 163.86, 164.78, 165.42

HR-MS (m/z): calcd. for C_28_H_29_N_9_O_4_S [L+H]^+^: 588.2132; found: 588.2136; Anal. calcd. (%) for C_28_H_29_N_9_O_4_S: C:57.23, H:4.97, N:21.45, found: C:56.99, H:4.82, N:21.25,

**6b:** 6-butyl-3,5,7-trimethyl-1-[[2-[2-oxo-2-(4-pyrimidin-2-ylpiperazin-1-yl)ethyl]sulfanyl-1,3,4-oxadiazol-5-yl]methoxy]pyrrolo[3,4-*d*]pyridazin-4-one

Whitish solid, Yield: 72,47%; m.p.: 198–200 °C

FT-IR (selected lines, γ_max_, cm^−1^): 2954, 2931, 2918 (C-H aliph.), 1640 (C=N)

^1^H NMR (300 MHz, CDCl_3_): δ= 0.95–1.00 (m, 3H, -CH_2_-CH_2_ CH_2_-CH_3_), 1.35-1.43 (m, 2H, -CH_2_-CH_2_-CH_2_-CH_3_), 1.62–1.67 (m, 2H, -CH_2_-CH_2_ CH_2_-CH_3_), 2.50 (s, 3H, 7-CH_3_), 2.69 (s, 3H, 5-CH_3_), 3.55 (s,3H, 3-CH_3_), 3.65-3.69 (m, 2H, CH_2_-piperazine), 3.74–3.77 (m, 2H, CH_2_-piperazine), 3.87–3.89 (m, 2H,-CH_2_-CH_2_-CH_2_-CH_3_), 3,91–3,98 (m, 2H, CH_2_-piperazine), 4.42 (s, 2H, S-CH_2_-), 5.45 (s, 2H, O-CH_2_), 6.57-6.60(t, 1H, ArH), 8.35–8.37 (m, 2H, ArH)

^13^C NMR (75 MHz, CDCl_3_): δ= 10.64, 11.19, 13.69, 20.04, 32.32, 37.02, 37.07, 42.18, 43.42, 43.68, 43.94, 45.92, 56.94, 108.15, 110.69, 111.85, 122.60, 129.13, 147.95, 157.77, 159.24, 161.07, 163.92, 164.81, 165.36

HR-MS (m/z): calcd. for C_26_H_33_N_9_O_4_S [L+H]^+^: 568.2460; found: 568.2449; Anal. calcd. (%) for C_26_H_33_N_9_O_4_S: C:55.01, H:5.86, N:22.21, found: C:55.27, H:5.91, N:22.15,

### 3.2. Cell Line

The study was conducted on a NHDF purchased from Lonza (Basel, Switzerland). These cells were a regular line of skin fibroblasts obtained from 54-years-old women.

Cells were grown at 37 °C in a humidified 5% CO_2_/95% air atmosphere incubator and passaged twice a week.

### 3.3. Cell Culture Media

The cells were cultured in a Dulbecco Modified Eagle Medium (DMEM) without phenol red, which was supplemented with 10% fetal bovine serum (FBS), 2 mM L-glutamine, 1.25 µg/mL amphotericin B, and 100 µg/mL gentamicin. Prepared culture medium was stored at 4–8 °C until full exploit, but not longer than a month.

### 3.4. Tested Compounds

Investigated derivatives of pyrrolo[3,4-*d*]pyridazinone were received from the Department of Chemistry of Drugs at Wroclaw Medical University. The compounds were dissolved with DMSO to a final concentration of 1mM. All prepared stock solutions were left at –20 °C for up to 6 months. Stock solutions were dissolved in the culture medium to accomplish the final concentrations of 100 µM, 50 µM and 10 µM for each compound.

### 3.5. Experimental Design

Cells were seeded into 96-well culture plates at a density of 10,000 cells/well, except fast halo assay (FHA) assay, in which cells were seeded into 24-well culture plates at the density of 25,000 cells/well. The cells were then allowed to adhere overnight. After this time, the medium was removed, and tested compounds were added for a further 24 h of incubation in 5% CO_2_, 95% humidity, 37 °C in MTT assay. Subsequently, the supernatant was removed, then the cells were washed with PBS, and the MTT assay was performed. For other assays, in the first step, the cells were incubated with the 100 µM H_2_O_2_ for 1h, then the supernatant was removed, and the cells were washed, and the cells were treated with tested compounds for the next 1 h.

The study included 2 control samples. A negative control, used as a reference, was a cell culture incubated in medium but without tested compounds. The positive control was used in the FHA and the level of oxygen free radicals and nitric oxide, which was a 1-h incubation of NHDF cells with 100 μM H_2_O_2_ without tested compounds.

Cell viability was assessed by metabolic activity in MTT assay, oxygen free radicals in DCF-DA assay and nitric oxide levels in Griess assay. FHA assay, which was used as well, allowed to estimate the number of DNA double-strand breaks (DSBs). The Cayman COX Inhibitor Screening Assay measured COX inhibition activity.

### 3.6. MTT Assay

The MTT assay was used to estimate the effects of investigated compounds on the metabolic activity of NHDF cells. The study started with the incubation of a proper cell line with tested compounds for 24 h at 37 °C. After that, the supernatant was replaced with 1 mg/mL MTT solution dissolved in MEM and plates were left for 2 h at 37 °C. Subsequently, the medium was removed, and for the next 30 min, the formazan crystals were dissolving in 100 µl of isopropanol. Absorbance was measured with Varioskan LUX microplate reader (Thermo Scientific, Waltham, MA, USA) at a wavelength of 570 nm.

### 3.7. Level of Reactive Oxygen Species

The DCF-DA assay was used to evaluate the level of ROS. The NHDF cells were cultured with 100 µM H_2_O_2_ to induce exogenous stress. Then, the solution was removed, cells were washed, and compounds were added for 1 h, and afterward, the medium was removed. The cells were washed with PBS, and 25 µM of DCF-DA solution dissolved in MEM without serum and phenol red was added. At this point, cultures were incubated for 1 h at 37 °C. The level of ROS was determined by fluorescence excitation at 485 nm and emission at 535 nm using a Varioskan LUX microplate reader (Thermo Scientific, Waltham, MA, USA).

### 3.8. Griess Assay

The Griess assay allows the establishment of nitric oxide production in the NHDF cell line. The Greiss reagent is a 1:1 mixture of 1% sulfanilamide in 5% phosphoric acid and 0.1% N-(1-Naphthyl)ethylenediamine dihydrochloride had to be prepared promptly before use.

After treatment with 100 µM H_2_O_2_, cell cultures were incubated with tested compounds for 1 h, and after that, 50 µl of the supernatant solution was moved into a new plate, while 50 µl of a Griess reagent was added and then left for the next 20 min at RT, in the darkness. Absorbance was measured at 548 nm with a Varioskan LUX microplate reader (Thermo Scientific, Waltham, MA, USA).

### 3.9. Fast Halo Assay

DSBs (DBSBs) in DNA were evaluated via the FHA. After incubating for 24 h with tested compounds (due to procedures previously described), cells were separated from the plate surface using TrypLE solution for 3 min. In the next step, cells were moved into the tube to inactivate the TrypLE solution, an equivalent quantity of medium was added. After cell centrifugation at 1000× *g* for 5 min and removing the supernatant, the cell pellet was washed using PBS and again centrifuged as previously. Afterward, the supernatant was removed, and the cells were suspended in PBS within a density of 1000 cells per 1 µl. In the next step, the tubes filled with cell suspension was placed in a water bath, and 120 ul of 1.25% low melting agarose in PBS was added. The obtained mixture was promptly compressed between a coverslip and an agarose-coated slide (high melting point) for 10 min. After that time, when gel formation on the cooling block was observed, coverslips were removed, while slides were put in the lysis buffer and left for 24h. The slides were moved afterward into an alkaline solution (pH = 13) for 30 min and, after that time, put twice in neutralizing buffer to wash. The slides were colored by 5 µM DAPI within 20 min and promptly evaluated using a fluorescence microscope. Having taken the photos, the cell nucleus diameter to halo diameter ratio was studied as a degree of DNA damage.

### 3.10. Fluorescence Spectroscopic Studies

Spectroscopic fluorescence studies were performed using a Cary Eclipse 500 spectrophotometer (Agilent, Santa Clara, CA, USA). A concentration of BSA was 5.0 × 10^−6^ mol·dm^−3^. A solution of BSA was titrated by successive additions 1.0 × 10^−3^ mol·dm^−3^ solution of studied compounds to give a final concentration 0.2 × 10^−6^–2.0 × 10^−6^ mol·dm^−3^. Experiments were carried out at three temperatures: 294, 301, and 308 K in pH = 7.4. Quenching spectra were recorded at excitation and an emission wavelength of 280 nm and 300–500 nm. The molar ratio compound/BSA was 0.1–2.0 with 0.2 steps. Binding site identification studies were indicated in the presence of the two site markers, phenylbutazone (PHB) and ibuprofen (IBP), as sites I and II markers, respectively. Concentrations of BSA and site markers were set at 1.0 × 10^−6^ and 3.0 × 10^−6^ mol dm^−3^, respectively.

### 3.11. Molecular Docking

The ground-state structures were calculated using density functional theory (DFT) with Becke’s three-parameter hybrid exchange function with the Lee-Yang–Parr gradient corrected correlation (B3LYP) [51,52,53] functional in combination with 6–311+G (d,p) basis set. Calculations were carried out using the Gaussian 2016 A.03 software package [54]. From the Protein Data Bank (http://www.rcsb.org), the following crystal structure was selected for docking studies: 4O1Z, 4M11, 3V03. The ligand and receptor files were prepared using AutoDock 4.2.6 software and AutoDock Tools 1.5.6. All the ligands and water molecules were removed, and then polar hydrogen atoms and Kollman charges were added to the protein structure. To prepare the ligand molecules, partial charges were calculated, nonpolar hydrogens were merged, and rotatable bonds were assigned. The interactions with COX-1, COX-2 and BSA were performed using AutoDock Script downloaded from The Scripps Research Institute (TSRI). The centers of grid boxes for COX-1 and COX-2 were set according to the Meloxicam binding site in the crystal structure 4O1Z, 4M11. The centers of grid boxes for BSA were set according to the binding site I phenylbutazone (PDB ID: 2BXC) and site II ibuprofen (PDB ID: 2BXG) on HSA [46]. The Lamarckian genetic algorithm was selected for the conformational search. The running times of the genetic algorithm and the evaluation times were set to 250 and 2.5million, respectively. After the molecular docking, the ligand-receptor complexes were further analyzed using Discovery Studio software (http://accelrys.com/).

### 3.12. Circular Dichroism

CD spectra were measured on Jasco J-1500 magnetic circular dichroism spectrometer. Spectra were collected in the range of 205–250 nm at a scan rate speed of 50 nm/min with 1 s response time and 10 mm path length. All spectra were measured once, each measurement with triple accumulation, and they were baseline corrected. All measurements for BSA solutions were made at RT under simulated physiological conditions in pH 7.4 in the absence and presence of analyzed compounds. Phosphate buffer was the solvent. Concentrations of BSA and analyzed compounds were: 1 × 10^−6^ mol/dm^3^ for BSA and 1 × 10^−3^ mol/dm^3^ for 5b, 6b, 5a, and 6a. Experiments were performed for BSA to each analyzed compound in molar ratios from 1:0 to 1:10.

### 3.13. Statistical Analysis

All results are presented as mean ± SEM (standard error of the mean) relative to the control (E/E_0_), where E is the culture with the tested substance, and E_0_ is the negative control (without H_2_O_2_ and tested compounds) or positive control (with H_2_O_2_ but without tested compounds). Statistical significance was calculated compared to the positive control for DCF-DA, Griess, and FHA assays or negative control for MTT assay.

The data have a normal distribution, so the ANOVA was used (with Tukey post-hoc tests). The significance level was set at *p* < 0.05. The relationship between DNA DSBs and free radical levels was shown by calculating Pearson correlation coefficients.

## 4. Conclusions

According to performed experiments, new derivatives of pyrrolo[3,4-*d*]pyridazinone exert promising anti-inflammatory and anti-oxidant activity. On the basis of our previous work [20] and inspired by Dogruer’s theory [29,30], we have decided to introduce the elongated, flexible 2-oxoethyl linker between 1,3,4-oxadiazole-2-thione ring and arylpiperazine moiety. The design of new compounds is therefore based on the double pharmacophore approach. We have combined in the structure of final derivatives the 1,3,4-oxadiazole moiety, which is one of the most important in medicinal chemistry and arylpiperazine pharmacophore, which can be distinguished in many potent anti-inflammatory agents [17,18,20,22,27,28,34,55]. Biological evaluation and molecular docking study clearly indicate that performed structural modification resulted in the good affinity of some of the new compounds to COX-2 isoenzyme and lack of activity towards COX-1. However, the inhibition activity is lower than that of Meloxicam, investigated structures take a position in the active site of COX-2 very similar to that reference drug. Probably, further structural optimization and changes in the type of arylpiperazine pharmacophore may result in enhanced COX-2 inhibitory activity. Nevertheless, what is worth to emphasize, the results of the COX Colorimetric Inhibitor Screening Assay stay in very good correlation with molecular docking. Furthermore, the compound which showed the best activity towards COX-2 in biological evaluation—**6a** is characterized by the lowest values of K_i_ when considering molecular docking study. As was mentioned before, there are slight structural differences between both cyclooxygenase isoforms, and the binding pocket of COX-2 is bigger than that of COX-1. It could explain the fact that the introduction of elongated 2-oxoethyl linker results in COX-2 selectivity of new derivatives. The fact that arylpiperazine pharmacophore is connected with 1,3,4-oxadiazole ring via sulfur, not nitrogen atom as formerly [20], could also impact the activity and cyclooxygenase selectivity of new compounds.

Promising anti-oxidant activity of new derivatives may suggest an additional mechanism of action involved in their anti-inflammatory activity. Needless to say, inflammation is firmly correlated with oxidative stress, and both processes can potentiate one another. In inflammatory cells, an excessive amount of ROS can be stated. This, in turn, promotes oxidative stress, which can cause oxidative damage, and in consequence, potentiate inflammation [56,57,58]. Therefore, the ability of scavenging free radicals and DNA protection properties can be very useful in the context of therapy of different inflammatory disorders.

Finally, both molecular docking study and spectroscopic investigations indicate that title compounds bind to BSA in a moderate manner and form complexes in a one-to-one ratio. According to performed experiments, investigated derivatives interact with BSA by means of complex formation. The favorable binding site of BSA is the hydrophobic cavity in site II (subdomain IIIA). According to CD results, the compounds **5a**,**b** caused greater changes of α-helix(%), which may suggest stronger binding of derivatives bearing phenylpiperazine pharmacophore. Such a result may indicate the potential long half-life on title compounds in vivo.

Summarizing, novel derivatives of pyrrolo[3,4-*d*]pyridazinone exert satisfactory anti-inflammatory and anti-oxidant activity. All compounds present selective inhibition activity towards COX-2 isoenzyme in both biological and molecular docking studies. Moreover, investigated structures possess the ability to scavenge reactive oxygen and nitrogen species. Such properties make the novel pyrrolo[3,4-*d*]pyridazinone derivatives the promising agents in the context of the development of new potent and safe analgesic and anti-inflammatory drug-candidates.

## Figures and Tables

**Table 1:** IC_50_ values determined for COX-1 and COX-2 enzymes; data are presented as mean and SD (standard deviation).

Compound	Cyclooxygenase Inhibition Assay IC50 [µM]	
	COX-1	COX-2
5a	ND ^1^	658.7 (15.0)
5b	ND ^1^	257.4 (11.3)
6a	ND ^1^	160.2 (6.8)
6b	ND ^1^	371.0 (10.1)
Meloxicam	83.7 (1.8)	59.2 (2.4)

^1^ ND—not detectable at tested concentrations.

**Table 2:** Energies of the binding complexes **5a, 6a, 5b, and 6b** with COX-2 obtained from molecular docking.

	∆G° [kJmol^−1^]	∆E1 [kJmol^−1^]	∆E2 [kJmol^−1^]	∆E3 [kJmol^−1^]	K_i_ [μM]
5a	−20.44	−30.18	−30.43	0.25	285.75
5b	−26.75	−39.20	−38.87	−0.33	20.39
6a	−30.54	−40.52	−40.88	−0.37	4.44
6b	−21.06	−33.52	−35.69	2.13	202.61
Meloxicam	−34.02	−37.74	−37.32	−0.42	1.09

ΔG°—binding free energy; ΔE_1_—intermolecular interaction energy, which is the sum of van der Waals energy, hydrogen bonding energy, desolvation free energy and electrostatic energy; ΔE_2_—the sum of van der Waals energy, hydrogen bonding energy and desolvation free energy; ΔE_3_—electrostatic energy, K_i_—inhibit constans.

**Table 3:** Reactive oxygen species (ROS) and reactive nitrogen species (RNS) scavenging activity of tested compounds and an impact on the DNA damage (*n* = 3); the results were compared to the positive control and expressed as E/E_0_ ratios; statistical significance calculated with post-hoc test compared to control (* *p* < 0.05; E0—culture incubated with 100 µM H_2_O_2_ but without test compounds); data are presented as mean and SEM.

Compound	Concentration [µM]	ROS	RNS	DNA Damage
	10	−33.1 (0.04) *	−3.7 (0.03)	−68 (0.1) *
5a	50	−34.2 (0.06) *	−5.9 (0.02)	−47 (0.08) *
	100	−28.7 (0.03) *	12.1 (0.02) *	−38 (0.1) *
	10	−29.0 (0.02) *	−6.7 (0.03) *	−59 (0.08) *
5b	50	−9.7 (0.02) *	3.4 (0.04)	−12 (0.07) *
	100	−0.5 (0.04)	9.8 (0.04)	5.7 (0.2)
	10	−7.4 (0.03)	−11.0 (0.07) *	−2.4 (0.1)
6a	50	12.3 (0.05)	−6.9 (0.05)	26 (0.06) *
	100	18.9 (0.06) *	−4.1 (0.04)	24 (0.08) *
	10	−24.1 (0.05) *	−7.9 (0.05) *	−70 (0.06) *
6b	50	−12.6 (0.04) *	−1.4 (0.04)	−22 (0.09) *
	100	−1.8 (0.02)	4.6 (0.02)	−9.7 (0.1) *

**Table 4:** Pearson correlation coefficients between DNA damage and ROS or NO levels.

Compound	ROS Level vs. DNA Damage	NO Level vs. DNA Damage
5a	0.589	0.650
5b	0.998	0.991
6a	0.953	0.888
6b	0.951	0.950

**Table 5:** The Stern–Volmer constant K_SV_ and the quenching rate constant k_q_, binding constants K_b_ and number of binding sites n, thermodynamic parameters for the interaction of BSA with studied compounds at different temperatures.

		Quenching		Binding			Thermodynamic		
	T [K]	K_sv_ × 10^5^ [dm^3^·mol^−1^]	k_q_ × 10^13^ [dm^3^·mol^−1^·s^−1^]	logK_b_	K_b_ × 10^4^ [dm^3^·mol^−1^]	n	∆G° [kJmol^−1^]	∆H° [kJmol^−1^]	∆S° [Jmol^−1^K^−1^]
**5a**	294	4.44	4.44	5.09 ± 0.14	12.30	0.91 ± 0.02	−29.31	−133.03	−352.78
	301	2.45	4.45	4.90 ± 0.30	7.94	0.92 ± 0.05			
	308	2.09	2.09	4.01 ± 0.13	1.02	0.77 ± 0.03			
**5b**	294	4.44	4.44	5.09 ± 0.14	12.30	0.91 ± 0.02	−29.31	−133.03	−352.78
	301	2.45	4.45	4.90 ± 0.30	7.94	0.92 ± 0.05			
	308	2.09	2.09	4.01 ± 0.13	1.02	0.77 ± 0.03			
**6a**	294	2.12	2.12	4.86 ± 0.05	7.24	0.92 ± 0.01	−27.51	−108.79	−276.43
	301	1.27	1.17	4.50 ± 0.13	3.16	0.89 ± 0.02			
	308	1.13	2.74	3.98 ± 0.27	0.96	0.81 ± 0.04			
**6b**	294	2.90	2.90	4.93 ± 0.07	8.51	0.91 ± 0.01	−27.37	−77.15	−169.33
	301	1.80	1.80	4.41 ± 0.29	2.57	0.85 ± 0.05			
	308	1.74	4.43	4.31 ± 0.26	2.04	0.84 ± 0.04			

**Table 6:** Energies of the binding complexes BSA with **5a, 5b, 6a, and 6b** obtained from molecular docking.

	Binding Site	∆G° [kJmol^−1^]	∆E_1_ [kJmol^−1^]	∆E_2_ [kJmol^−1^]	∆E_3_ [kJmol^−1^]
5a	site I	−31.43	−41.42	−40.88	−0.54
	site II	−38.96	−48.94	−48.44	−0.46
5b	site I	−27.38	−39.83	−38.70	−1.13
	site II	−38.87	−51.37	−51.03	−0.33
6a	site I	−34.73	−44.72	−40.34	−4.39
	site II	−36.94	−47.10	−44.51	−2.59
6b	site I	−30.55	−43.01	−38.70	−4.31
	site II	−39.12	−51.62	−49.49	−0.46

ΔG°—binding free energy; ΔE_1_—intermolecular interaction energy, which is the sum of van der Waals energy, hydrogen bonding energy, desolvation free energy and electrostatic energy; ΔE_2_—the sum of van der Waals energy, hydrogen bonding energy and desolvation free energy; ΔE_3_—electrostatic energy.

**Table 7:** The values of calculated α-helix(%) for BSA with the absence and presence of all analyzed compounds.

BSA/Analyzed Compound Molar Ratio	α-helix(%)			
	5a	5b	6a	6b
1:0	**54.05**	**53.55**	**53.78**	**53.46**
1:0.5	51.08	52.15	52.77	52.45
1:1	50.51	51.11	51.72	51.83
1:2	49.94	50.50	51.70	51.26
1:3	49.70	50.47	51.45	50.91
1:4	49.62	49.81	51.49	50.47
1:5	49.48	49.78	51.40	50.23
1:6	48.85	49.51	50.28	50.15
1:7	48.57	49.21	50.29	50.10
1:8	48.23	48.85	50.31	49.97
1:9	48.11	48.06	50.24	49.82
1:10	**47.59**	**47.76**	**50.11**	**49.76**

Numbers in bold represent the initial and final values of α-helical content of BSA in carried out experiment.

## Supplementary Materials

The following are available online at https://www.mdpi.com/1422-0067/21/24/9623/s1.

## References

[1] Marnett L.J. (2000). Cyclooxygenase mechanisms. Curr. Opin. Chem. Biol..

[2] Blobaum A.L., Marnett L.J., Hancock A.B. (2007). PerspectiVe Structural and Functional Basis of Cyclooxygenase Inhibition. J. Med. Chem..

[3] Vane J.R., Botting R.M. (1998). Mechanism of action of nonsteroidal anti-inflammatory drugs. Am. J. Med..

[4] Smith W.L., Urade Y., Jakobsson J. (2011). Enzymes of the Cyclooxygenase Pathways of Prostanoid Biosynthesis. Chem. Rev.

[5] Cashman J.N. (1996). The Mechanisms of Action of NSAIDs in Analgesia. Drugs.

[6] Leuti A., Fazio D., Fava M., Piccoli A., Oddi S., Maccarrone M. (2020). Bioactive lipids, inflammation and chronic diseases. Adv. Drug Deliv. Rev..

[7] Sostres C., Gargallo C.J., Arroyo M.T., Lanas A. (2010). Adverse effects of non-steroidal anti-inflammatory drugs (NSAIDs, aspirin and coxibs) on upper gastrointestinal tract. Best Pract. Res. Clin. Gastroenterol..

[8] Bidaut-Russell M., Gabriel S.E. (2001). Adverse gastrointestinal effects of NSAIDs: Consequences and costs. Best Pract. Res. Clin. Gastroenterol..

[9] Laine L. (2003). Gastrointestinal effects of NSAIDs and coxibs. J. Pain Symptom Manag..

[10] Wallace J.L., Devchand P.R. (2005). Emerging roles for cyclooxygenase-2 in gastrointestinal mucosal defense. Br. J. Pharmacol..

[11] Chandrasekharan N.V., Dai H., Lamar K., Roos T., Evanson N.K., Tomsik J., Elton T.S., Simmons D.L. (2002). COX-3, a cyclooxygenase-1 variant inhibited by acetaminophen and other analgesic/antipyretic drugs: Cloning, structure, and expression PCOX-1 proteins). COX-3 and one of the PCOX-1 proteins (PCOX-1a) are made from the COX-1 gene but retain intron 1 in their mRNAs. PCOX-1 proteins additionally contain an in-frame deletion of exons 5-8 of the COX-1 mRNA. COX-3 and PCOX mRNAs. Proc. Natl. Acad. Sci. USA.

[12] Soll A.H., McCarthy D. (1999). NSAID-related gastrointestinal complications. Clin. Cornerstone.

[13] Wallace J.L. (2012). NSAID gastropathy and enteropathy: Distinct pathogenesis likely necessitates distinct prevention strategies. Br. J. Pharmacol..

[14] García Rodríguez L.A., Barreales Tolosa L. (2007). Risk of Upper Gastrointestinal Complications Among Users of Traditional NSAIDs and COXIBs in the General Population. Gastroenterology.

[15] Takeuchi K. (2012). Pathogenesis of NSAID-induced gastric damage: Importance of cyclooxygenase inhibition and gastric hypermotility. World J. Gastroenterol..

[16] Esposito G., Pagano E., Ii F., Kaji I.I., Scarpignato C., Colucci R., Pellegrini C., Fornai M., Tirotta E., Antonioli L. (2018). Pathophysiology of NSAID-Associated Intestinal Lesions in the Rat: Luminal Bacteria and Mucosal Inflammation as Targets for Prevention. Front. Pharmacol..

[17] Koksal M., Ozkan-Dagliyan I., Ozyazici T., Kadioglu B., Sipahi H., Bozkurt A., Bilge S.S. (2017). Some Novel Mannich Bases of 5-(3,4-Dichlorophenyl)-1,3,4-oxadiazole-2(3H)-one and Their Anti-Inflammatory Activity. Arch. Pharm..

[18] El-Sayed N.A., Nour M.S., Salem M.A., Arafa R.K. (2019). New oxadiazoles with selective-COX-2 and EGFR dual inhibitory activity: Design, synthesis, cytotoxicity evaluation and in silico studies. Eur. J. Med. Chem..

[19] Banerjee A.G., Das N., Shengule S.A., Sharma P.A., Srivastava R.S., Shrivastava S.K. (2016). Design, synthesis, evaluation and molecular modelling studies of some novel 5,6-diphenyl-1,2,4-triazin-3(2H)-ones bearing five-member heterocyclic moieties as potential COX-2 inhibitors: A hybrid pharmacophore approach. Bioorg. Chem..

[20] Szczukowski Ł., Redzicka A., Wiatrak B., Krzyżak E., Marciniak A., Gębczak K., Gębarowski T., Świątek P. (2020). Design, synthesis, biological evaluation and in silico studies of novel pyrrolo[3,4-d]pyridazinone derivatives with promising anti-inflammatory and antioxidant activity. Bioorg. Chem..

[21] Wakulik K., Wiatrak B., Szczukowski Ł., Bodetko D., Szandruk-Bender M., Dobosz A., Piotr’ P., Atek P., Asiorowski K.G. (2020). Effect of Novel Pyrrolo[3,4-d]pyridazinone Derivatives on Lipopolysaccharide-Induced Neuroinflammation. Int. J. Mol. Sci. Artic..

[22] Malinka W., Redzicka A., Jastrzbska Wiȩsek M., Filipek B., Dybała M., Karczmarzyk Z., Urbańczyk-Lipkowska Z., Kalicki P. (2011). Derivatives of pyrrolo[3,4-d]pyridazinone, a new class of analgesic agents. Eur. J. Med. Chem..

[23] Mogilski S., Kubacka M., Redzicka A., Kazek G., Dudek M., Malinka W., Filipek B. (2015). Antinociceptive, anti-inflammatory and smooth muscle relaxant activities of the pyrrolo[3,4-d]pyridazinone derivatives: Possible mechanisms of action. Pharmacol. Biochem. Behav..

[24] Kohara Y., Imamiya E., Kubo K., Wada T., Inada Y., Naka T. (1995). A new class of angiotensin II receptor antagonists with a novel acidic bioisostere. Bioorganic Med. Chem. Lett..

[25] Kohara Y., Kubo K., Imamiya E., Wada T., Inada Y., Naka T. (1996). Synthesis and Angiotensin II Receptor Antagonistic Activities of Benzimidazole Derivatives Bearing Acidic Heterocycles as Novel Tetrazole Bioisosteres 1. J. Med. Chem..

[26] Tagad H.D., Hamada Y., Nguyen J.T., Hamada T., Abdel-Rahman H., Yamani A., Nagamine A., Ikari H., Igawa N., Hidaka K. (2010). Design of pentapeptidic BACE1 inhibitors with carboxylic acid bioisosteres at P1′ and P4 positions. Bioorganic Med. Chem..

[27] Palkar M.B., Singhai A.S., Ronad P.M., Vishwanathswamy A.H.M., Boreddy T.S., Veerapur V.P., Shaikh M.S., Rane R.A., Karpoormath R. (2014). Synthesis, pharmacological screening and in silico studies of new class of Diclofenac analogues as a promising anti-inflammatory agents. Bioorganic Med. Chem..

[28] Manjunatha K., Poojary B., Lobo P.L., Fernandes J., Kumari N.S. (2010). Synthesis and biological evaluation of some 1,3,4-oxadiazole derivatives. Eur. J. Med. Chem..

[29] Dogruer D.S., Sahin M.F., Ünlü S., Ito S. (2000). Studies on some 3(2H)-pyridazinone derivatives with antinociceptive activity. Arch. Pharm..

[30] Dogruer D.S., Kupeli E., Yesilada E., Sahin M.F. (2004). Synthesis of New 2-[1(2H)-Phthalazinon-2-yl]acetamide and 3-[1(2H)-Phthalazinon-2-yl]propanamide Derivatives as Antinociceptive and Anti-inflammatory Agents. Arch. Pharm..

[31] Dekhane D.V., Pawar S.S., Gupta S., Shingare M.S., Patil C.R., Thore S.N. (2011). Synthesis and anti-inflammatory activity of some new 4,5-dihydro-1,5- diaryl-1H-pyrazole-3-substituted-heteroazole derivatives. Bioorg. Med. Chem. Lett..

[32] Bansal S., Bala M., Suthar S.K., Choudhary S., Bhattacharya S., Bhardwaj V., Singla S., Joseph A. (2014). Design and synthesis of novel 2-phenyl-5-(1,3-diphenyl-1H-pyrazol-4-yl)-1, 3,4-oxadiazoles as selective COX-2 inhibitors with potent anti-inflammatory activity. Eur. J. Med. Chem..

[33] Gupta S., Pandey D., Mandalapu D., Bala V., Sharma V., Shukla M., Yadav S.K., Singh N., Jaiswal S., Maikhuri J.P. (2016). Design, synthesis and biological profiling of aryl piperazine based scaffolds for the management of androgen sensitive prostatic disorders. Medchemcomm.

[34] Świątek P., Strzelecka M., Urniaz R., Gębczak K., Gębarowski T., Gąsiorowski K., Malinka W. (2017). Synthesis, COX-1/2 inhibition activities and molecular docking study of isothiazolopyridine derivatives. Bioorg. Med. Chem..

[35] Chen G.Z., Huang X.Z., Xu J.H., Zneng Z.Z., Wang Z.B. (1990). The Methods of Fluorescence Analysis, 2ed ed..

[36] Wani T.A., Bakheit A.H., Zargar S., Bhat M.A., Al-Majed A.A. (2019). Molecular docking and experimental investigation of new indole derivative cyclooxygenase inhibitor to probe its binding mechanism with bovine serum albumin. Bioorg. Chem..

[37] Lakowicz J.R. (2006). Principles of Fluorescence Spectroscopy.

[38] Ware W.R. (1962). Oxygen quenching of fluorescence in solution: An experimental study of the diffusion process. J. Phys. Chem..

[39] Mohammadnia F., Fatemi M.H., Taghizadeh S.M. (2020). Study on the interaction of anti-inflammatory drugs with human serum albumin using molecular docking, quantitative structure–activity relationship, and fluorescence spectroscopy. Luminescence.

[40] Dufour C., Dangles O. (2005). Flavonoid-serum albumin complexation: Determination of binding constants and binding sites by fluorescence spectroscopy. Biochim. Biophys. Acta Gen. Subj..

[41] Abdelhameed A.S., Bakheit A.H., Mohamed M.S., Eldehna W.M., Abdel-Aziz H.A., Attia M.I. (2017). Synthesis and biophysical insights into the binding of a potent anti-proliferative non-symmetric bis-isatin derivative with bovine serum albumin: Spectroscopic and molecular docking approaches. Appl. Sci..

[42] Suryawanshi V.D., Walekar L.S., Gore A.H., Anbhule P.V., Kolekar G.B. (2016). Spectroscopic analysis on the binding interaction of biologically active pyrimidine derivative with bovine serum albumin. J. Pharm. Anal..

[43] Wani T.A., Bakheit A.H., Al-Majed A.R.A., Bhat M.A., Zargar S. (2017). Study of the interactions of bovine serum albumin with the new anti-inflammatory agent 4-(1,3-dsioxo-1,3-dihydro-2H-isoindol-2-yl)-N-[(4-ethoxy-phenyl) methylidene]benzohydrazide using a multi-spectroscopic approach and molecular docking. Molecules.

[44] Krzyżak E., Szkatuła D., Wiatrak B., Gębarowski T., Marciniak A. (2020). Synthesis, Cyclooxygenases Inhibition Activities and Interactions with BSA of N-substituted 1H-pyrrolo[3,4-c]pyridine-1,3(2H)-diones Derivatives. Molecules.

[45] Sudlow G., Birkett D.J., Wade D.N. (1975). The Characterization of Two Specific Drug Binding Sites on Human Serum Albumin. Mol. Pharmacol..

[46] Ghuman J., Zunszain P.A., Petitpas I., Bhattacharya A.A., Otagiri M., Curry S. (2005). Structural basis of the drug-binding specificity of human serum albumin. J. Mol. Biol..

[47] Klotz I.M., Urquhart J.M. (1949). The Binding of Organic Ions by Proteins. Effect of Temperature. J. Am. Chem. Soc..

[48] Kelly S., Price N. (2005). The Use of Circular Dichroism in the Investigation of Protein Structure and Function. Curr. Protein Pept. Sci..

[49] Kelly S.M., Jess T.J., Price N.C. (2005). How to study proteins by circular dichroism. Biochim. Biophys. Acta Proteins Proteom..

[50] Lu Z.X., Cui T., Shi Q.L. (1987). Applications of Circular Dichroism (CD) and Optical Rotatory Dispersion (ORD) in Molecular Biology.

[51] Becke A.D. (1993). Density-functional thermochemistry. III. The role of exact exchange. J. Chem. Phys..

[52] Lee C., Yang W., Parr R.G. (1988). Development of the Colle-Salvetti correlation-energy formula into a functional of the electron density. Phys. Rev. B.

[53] Perdew J.P., Wang Y. (1992). Accurate and simple analytic representation of the electron-gas correlation energy. Phys. Rev. B.

[54] Frisch M.J., Trucks G.W., Schlegel H.B., Scuseria G.E., Robb M.A., Cheeseman J.R., Scalmani G., Barone V., Petersson G.A., Nakatsuji H. (2016). Gaussian~16 {R}evision {A}.03 2016.

[55] Redzicka A., Szczukowski Ł., Kochel A., Wiatrak B., Gębczak K., Czyżnikowska Ż. (2019). COX-1/COX-2 inhibition activities and molecular docking study of newly designed and synthesized pyrrolo[3,4-c]pyrrole Mannich bases. Bioorg. Med. Chem..

[56] McGarry T., Biniecka M., Veale D.J., Fearon U. (2018). Hypoxia, oxidative stress and inflammation. Free Radic. Biol. Med..

[57] Gao Z., Zhang H., Liu J., Lau C.W., Liu P., Chen Z.Y., Lee H.K., Tipoe G.L., Ho H.M., Yao X. (2015). Cyclooxygenase-2-dependent oxidative stress mediates palmitate-induced impairment of endothelium-dependent relaxations in mouse arteries. Biochem. Pharmacol..

[58] Burdon C., Mann C., Cindrova-Davies T., Ferguson-Smith A.C., Burton G.J. (2007). Oxidative Stress and the Induction of Cyclooxygenase Enzymes and Apoptosis in the Murine Placenta. Placenta.

