# Supplementary material for: Design, Synthesis and Comprehensive Investigations of Pyrrolo[3,4-d]pyridazinone-Based 1,3,4-Oxadiazole as New Class of Selective COX-2 Inhibitors
Source: Int J Mol Sci. 2020 Dec 17;21(24):9623. doi: 10.3390/ijms21249623 (PMC7766220; doi:10.3390/ijms21249623)
Supplement: Supplementary file 1 [file ijms-21-09623-s001.pdf]

SUPPLEMENTARY DATA

Pyrrolo[3,4-*d*]pyridazinone derivatives bearing 1,3,4-oxadiazole moiety can serve as new class of selective COX-2 inhibitors. Design, synthesis, molecular docking, comprehensive spectroscopic and *in vitro* investigations

Łukasz Szczukowski[1*], Edward Krzyżak[2] Adrianna Zborowska[3], Patrycja Zając[3], Katarzyna Potyrak[3], Krzysztof Peregrym[3], , Benita Wiatrak[4, 5], Aleksandra Marciniak[2] and Piotr Świątek[1]

[1] *Department of Medicinal Chemistry, Wroclaw Medical University, Borowska 211, 50-556 Wroclaw, Poland;*
[2] *Department of Inorganic Chemistry, Wroclaw Medical University, Borowska 211a, 50-556, Wroclaw, Poland;*
[3] *Student Scientific Club of Medicinal Chemistry, Wroclaw Medical University, Borowska 211, 50-556 Wroclaw, Poland;*
[4] *Department of Basic Medical Sciences, Wroclaw Medical University, Borowska 211, 50-556 Wroclaw, Poland;*
[5] *Department of Pharmacology, Wroclaw Medical University, Mikulicza-Radeckiego 2 50-345 Wroclaw, Poland;*

* Correspondence: lukasz.szczukowski@umed.wroc.pl; Tel.: +48 71 784-03-90

Table of contents

1. NMR spectra of new compounds 2
2. IR spectra of new compounds 6
3. Mass spectra of new compounds 8
4. Figures and tables 10
Fig. S1. Equtions used spectroscopic studies 10

1. NMR spectra of new compounds

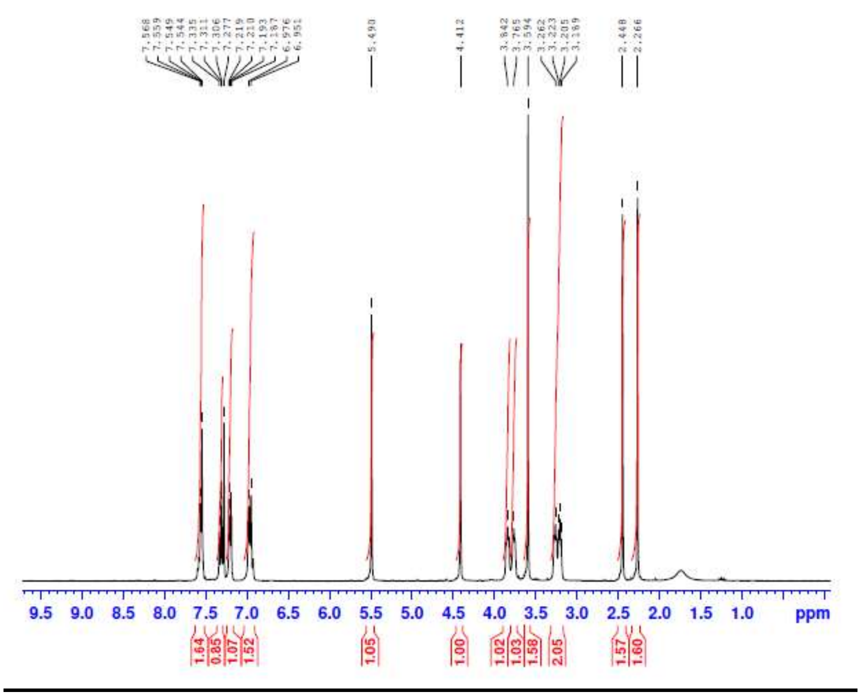

5a

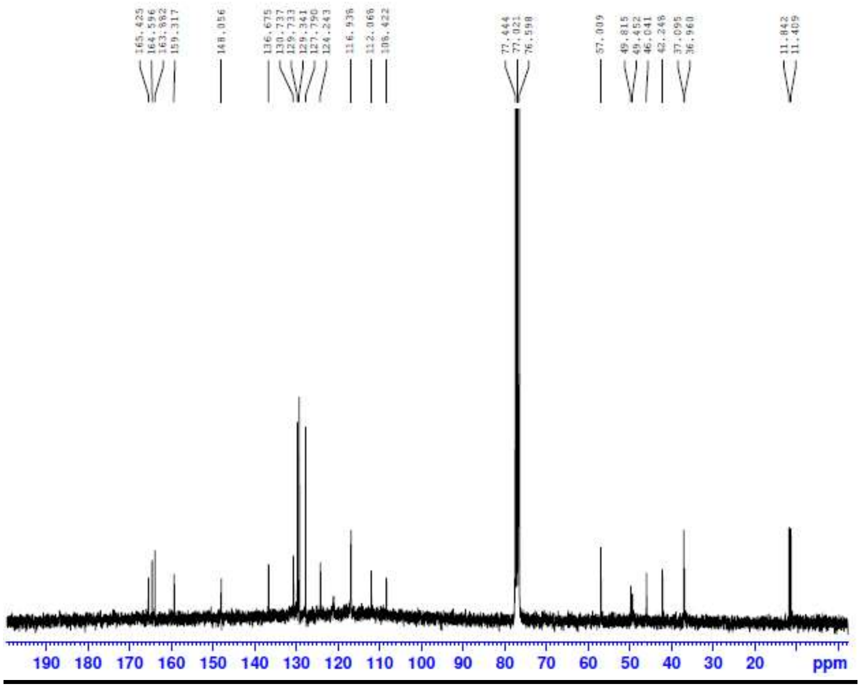

5a

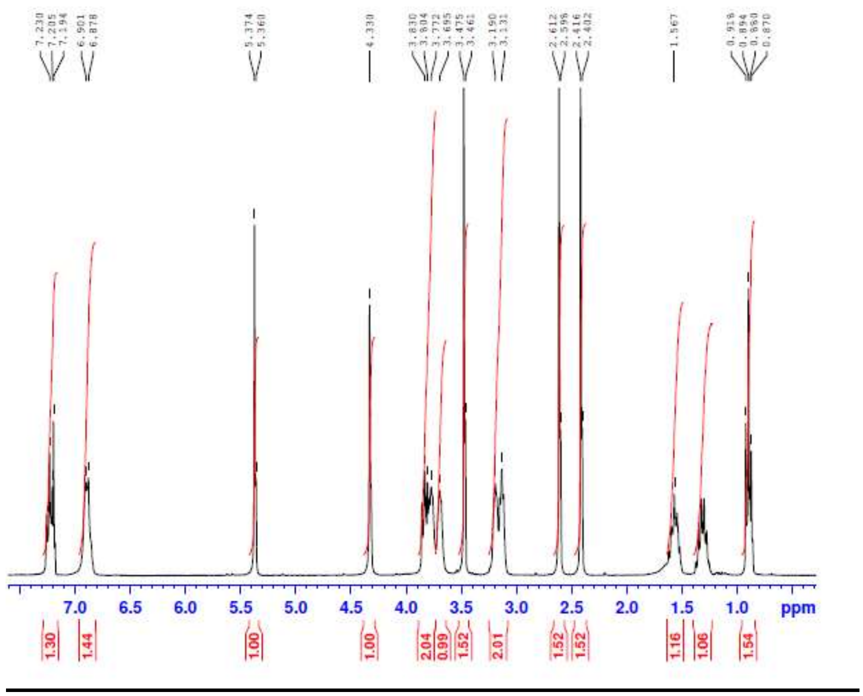

5b

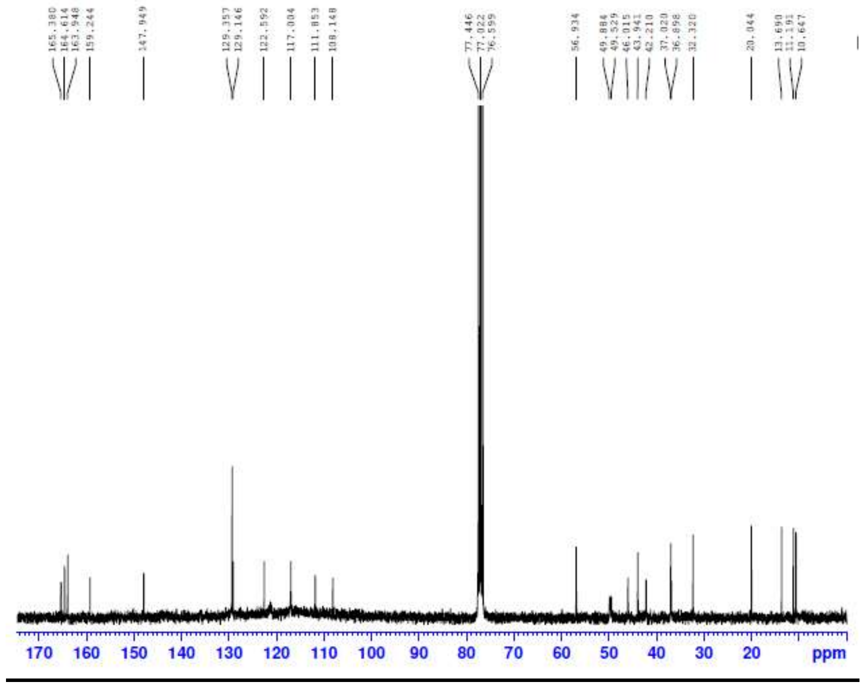

5b

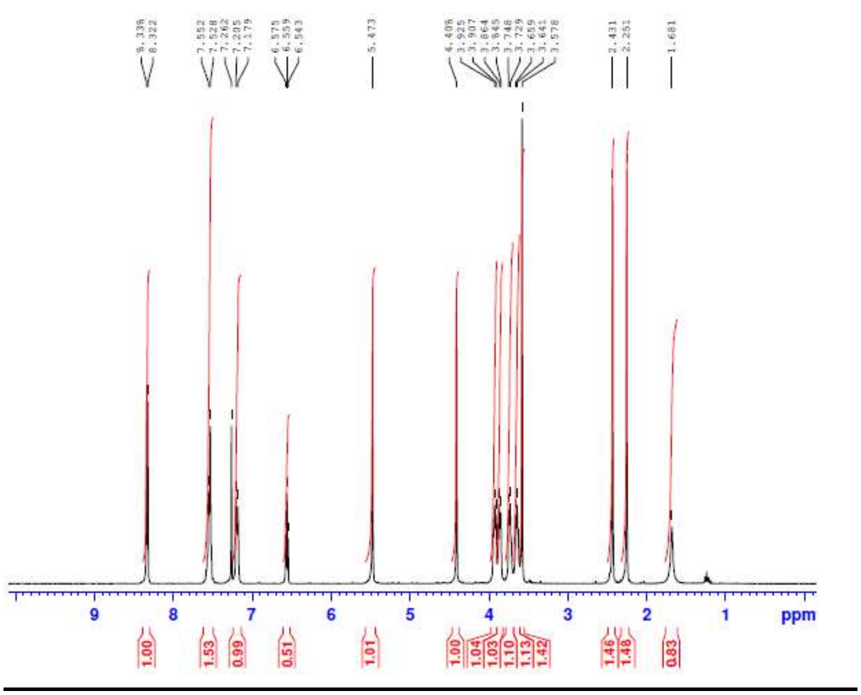
8.338
8.322
7.552
7.528
7.262
7.205
7.179
6.575
6.559
6.543
5.473
4.406
3.925
3.907
3.864
3.845
3.748
3.729
3.659
3.641
3.578
2.431
2.251
1.681
9 8 7 6 5 4 3 2 1 ppm
1.00
1.53
0.99
0.51
1.01
1.00
1.04
1.03
1.10
1.13
1.42
1.46
1.48
0.83

6a

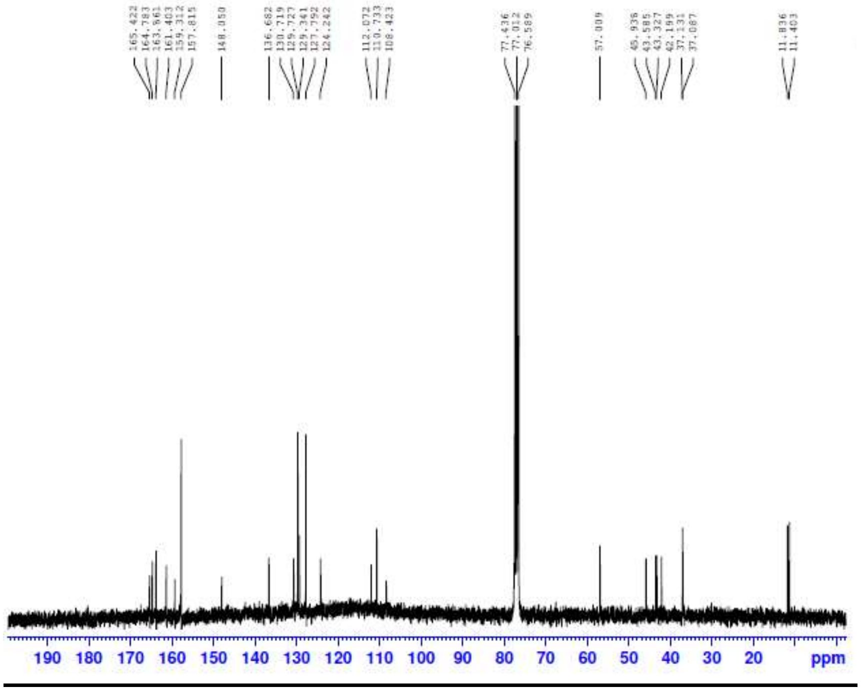
165.422
164.783
163.961
161.403
159.312
157.815
148.050
136.682
130.719
129.727
129.341
127.792
124.242
112.072
110.733
108.423
77.436
77.012
76.589
57.009
45.936
43.585
43.327
42.199
37.131
37.087
11.836
11.403
190 180 170 160 150 140 130 120 110 100 90 80 70 60 50 40 30 20 ppm

6a

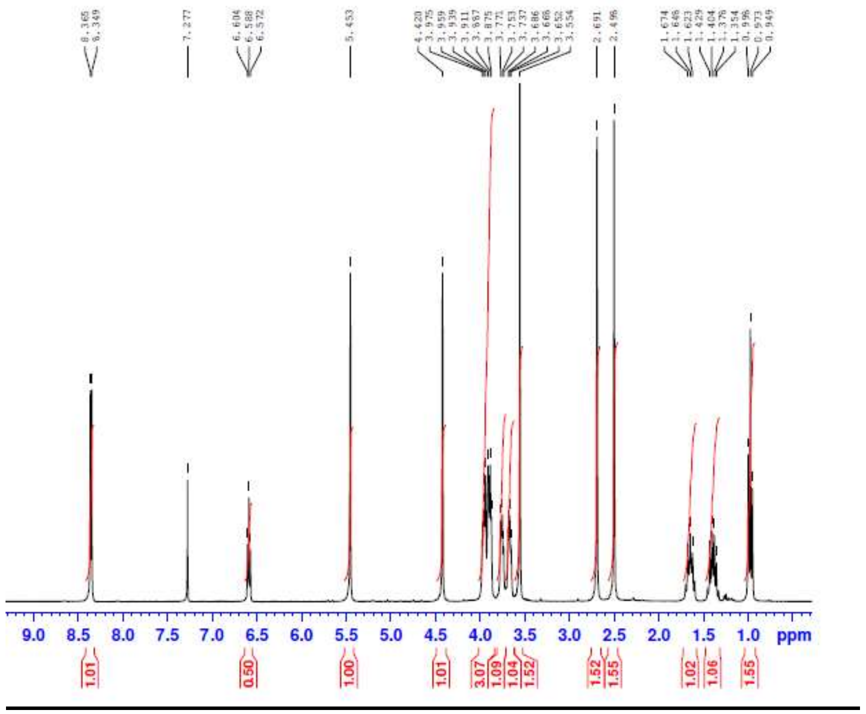
8.365
8.349
7.277
6.604
6.588
6.572
5.453
4.420
2.691
2.498
9.0 8.5 8.0 7.5 7.0 6.5 6.0 5.5 5.0 4.5 4.0 3.5 3.0 2.5 2.0 1.5 1.0 ppm
1.01
0.50
1.00
1.01
3.07
1.09
1.04
1.52
1.52
1.55
1.02
1.06
1.55

6b

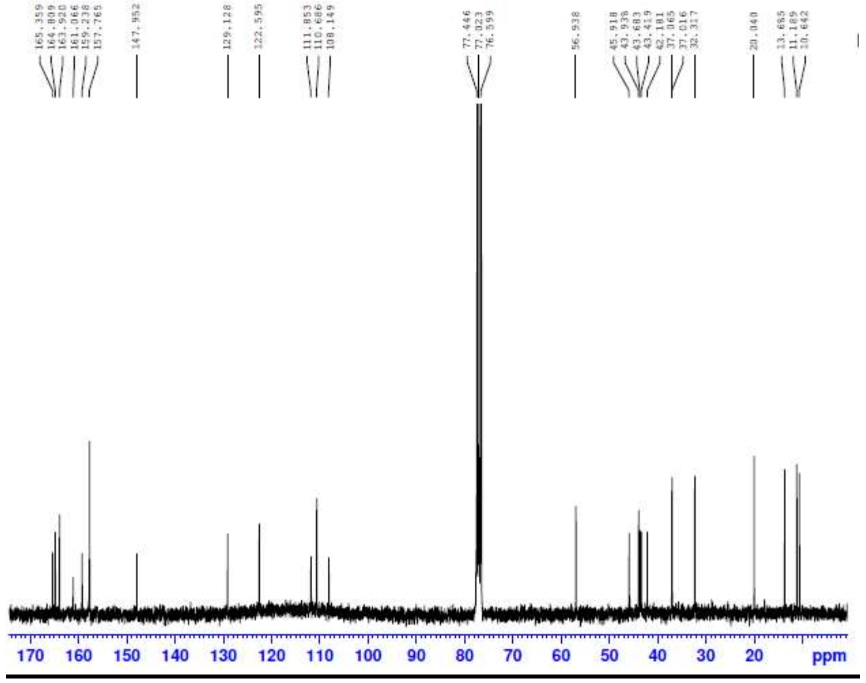
147.952
129.128
122.595
77.446
77.023
76.599
56.938
20.040
170 160 150 140 130 120 110 100 90 80 70 60 50 40 30 20 ppm

6b

2. IR spectra of new compounds

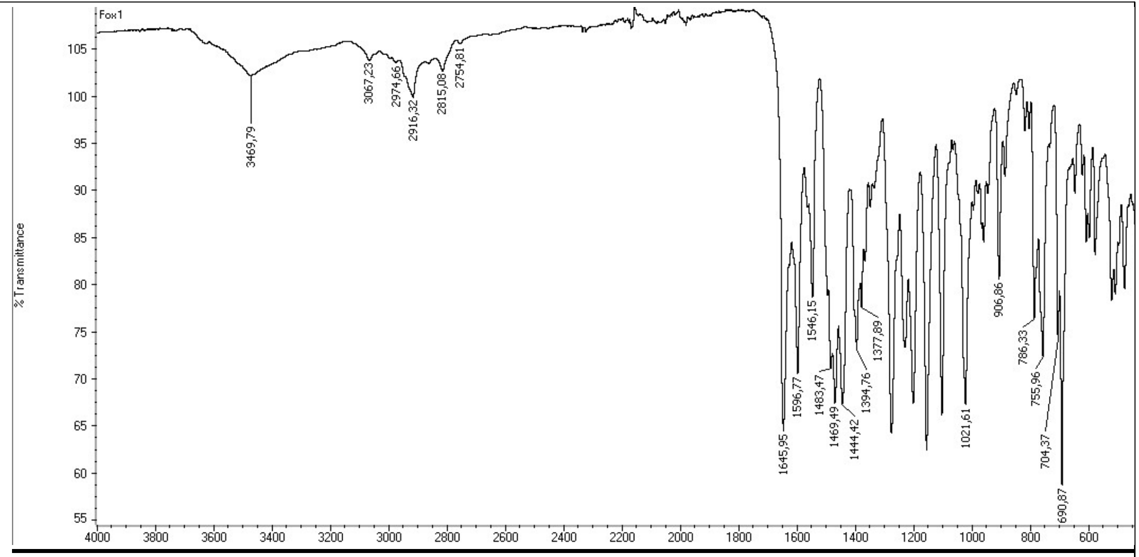

5a

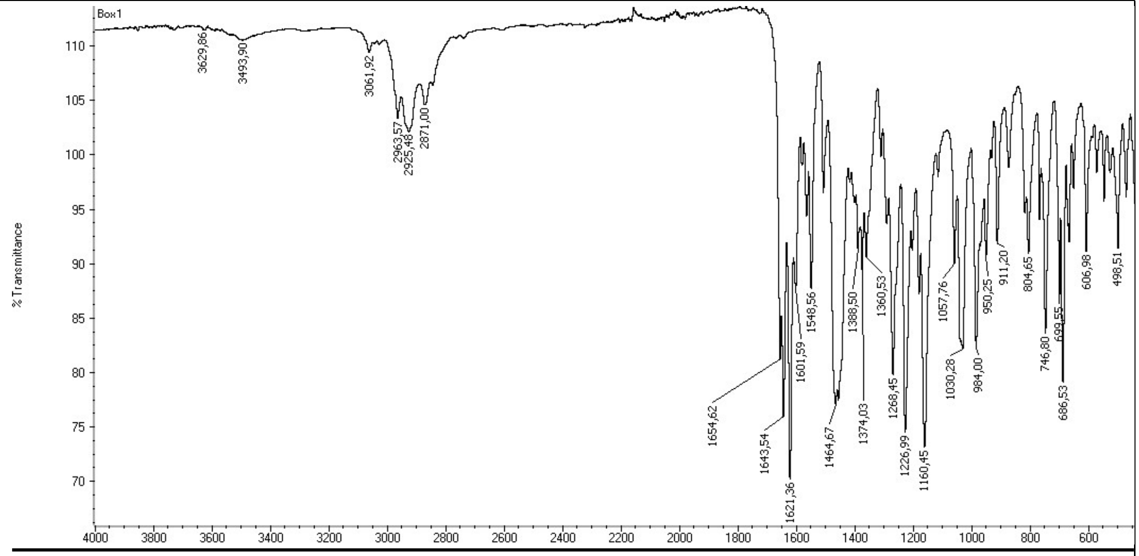

5b

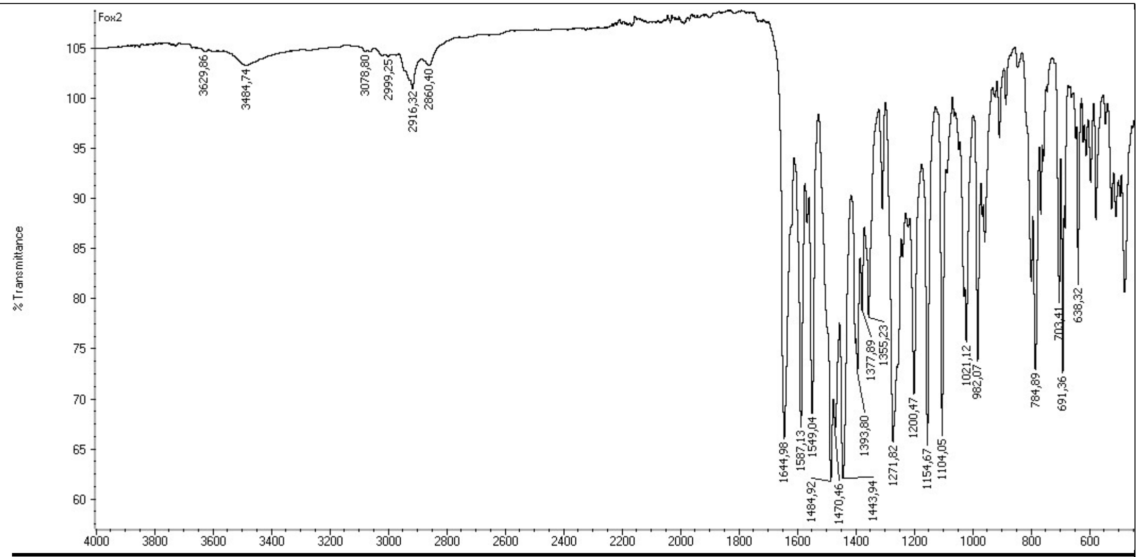
Fox2
% Transmittance
105
100
95
90
85
80
75
70
65
60
4000
3800
3600
3400
3200
3000
2800
2600
2400
2200
2000
1800
1600
1400
1200
1000
800
600
3629,86
3484,74
3078,80
2999,25
2916,32
2860,40
1644,98
1587,13
1549,04
1484,92
1470,46
1443,94
1393,80
1377,89
1355,23
1271,82
1200,47
1154,67
1104,05
1021,12
982,07
784,89
703,41
691,36
638,32

6a

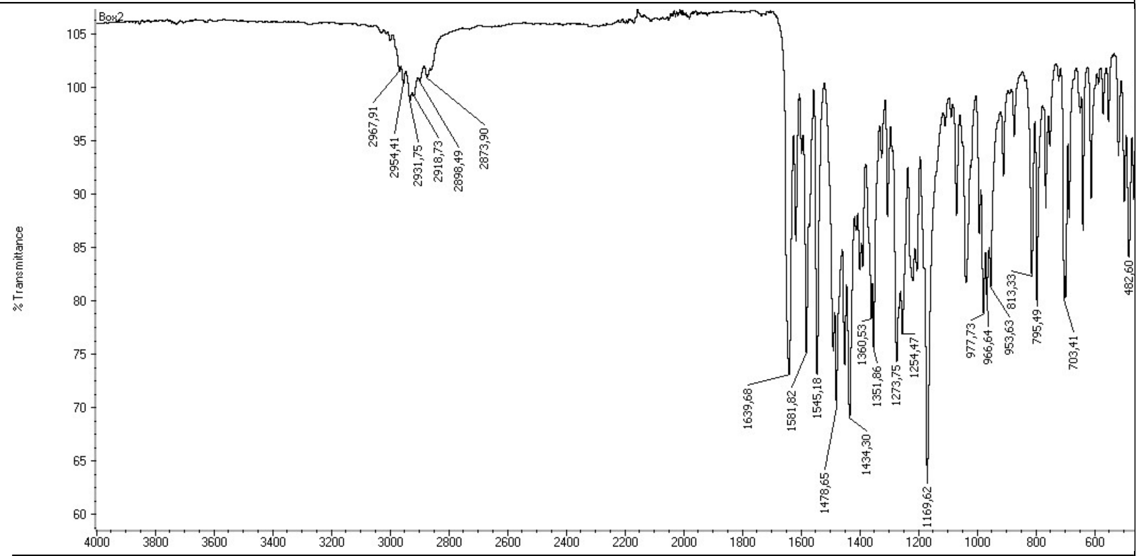
Box2
% Transmittance
105
100
95
90
85
80
75
70
65
60
4000
3800
3600
3400
3200
3000
2800
2600
2400
2200
2000
1800
1600
1400
1200
1000
800
600
2967,91
2954,41
2931,75
2918,73
2898,49
2873,90
1639,68
1581,82
1545,18
1478,65
1434,30
1360,53
1351,86
1273,75
1254,47
1169,62
977,73
966,64
953,63
813,33
795,49
703,41
482,60

6b

3. Mass spectra of new compounds

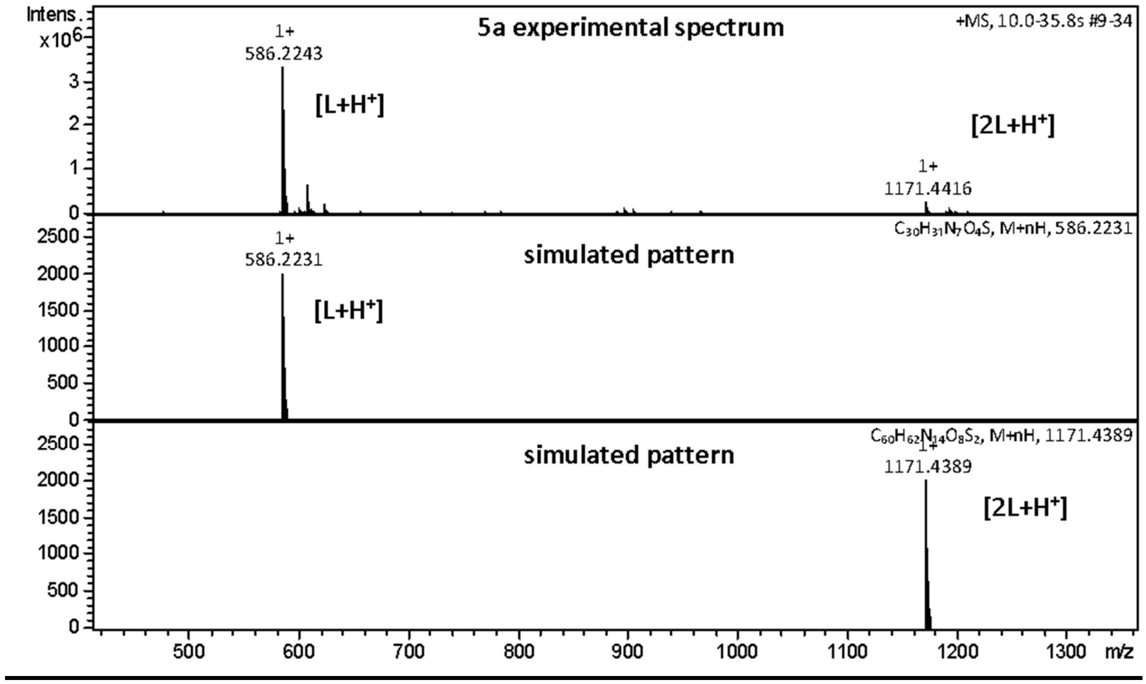

5a

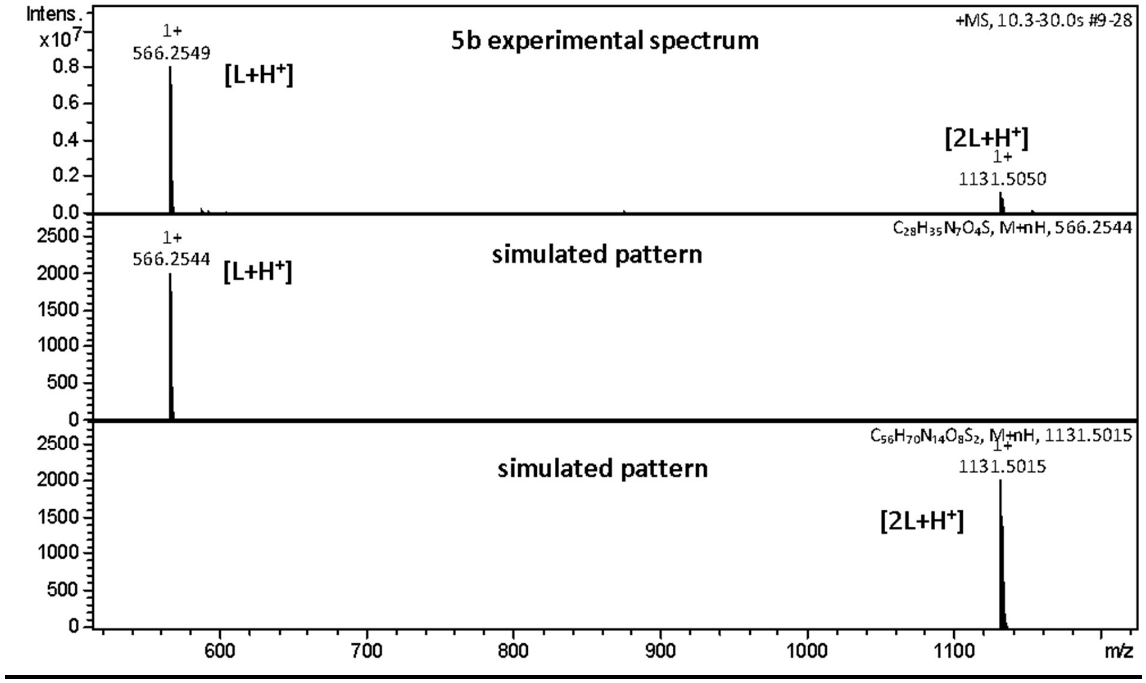

5b

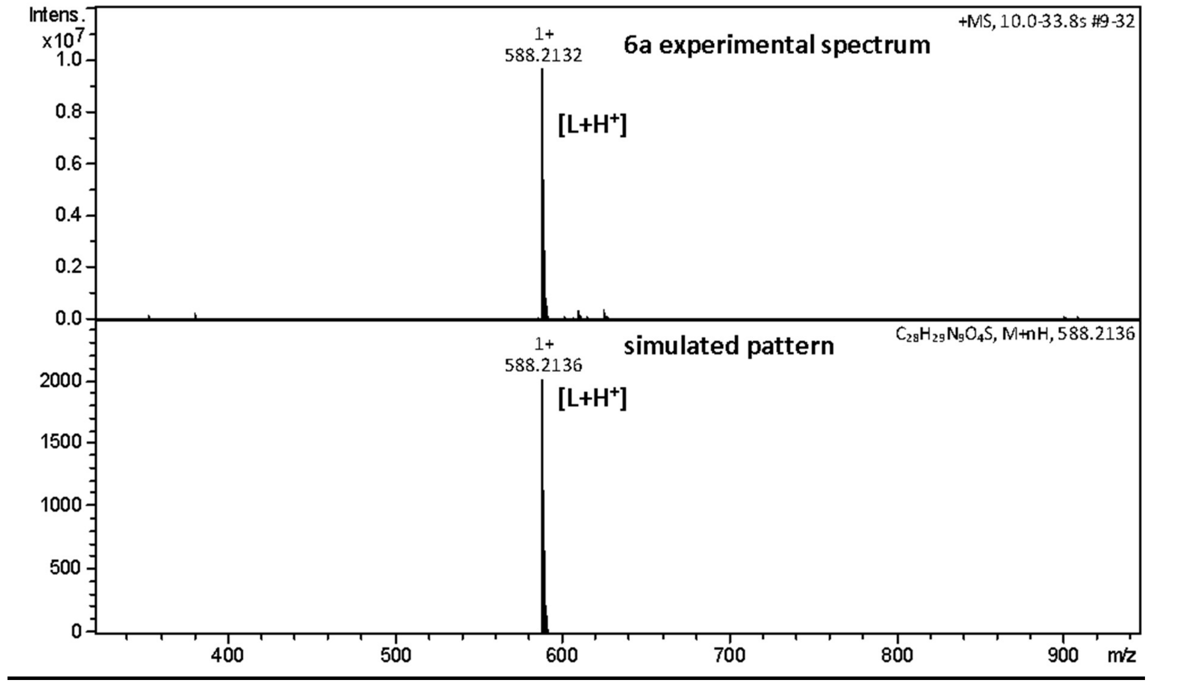
Intens.
x10^7
6a experimental spectrum
+MS, 10.0-33.8s #9-32
1+
588.2132
[L+H+]
simulated pattern
C28H29N9O4S, M+nH, 588.2136
1+
588.2136
[L+H+]
m/z

6a

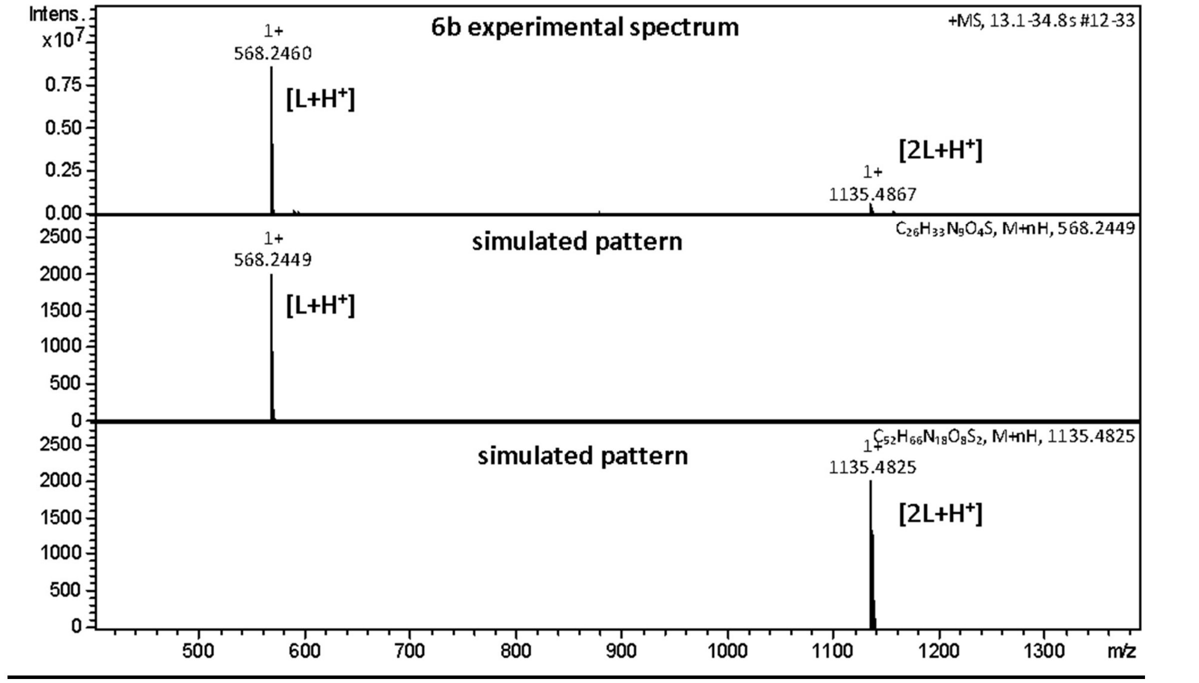
Intens.
x10^7
6b experimental spectrum
+MS, 13.1-34.8s #12-33
1+
568.2460
[L+H+]
[2L+H+]
1+
1135.4867
simulated pattern
C26H33N9O4S, M+nH, 568.2449
1+
568.2449
[L+H+]
simulated pattern
C52H66N18O8S2, M+nH, 1135.4825
1+
1135.4825
[2L+H+]
m/z

6b

4. Figures and tables

Fig. S1. Equtions used spectroscopic studies.

$$F_{corr} = F_{obs}10^{\frac{(A_{ex}+A_{em})}{2}}\ (1)$$
**Eq.1**<br>where, F_{corr} and F_{obs} are the corrected and observed fluorescence intensities, respectively. A_{ex} and A_{em} are the absorbance values at excitation and emission wavelengths, respectively.
$$\frac{F_O}{F} = 1 + k_q\tau[Q] = 1 + K_{SV}(2)$$
**Eq.2**<br>where F_0 and F are the steady-state fluorescence intensities at the maximum wavelength in the absence and presence of quencher, respectively, k_q the quenching rate constant of the biomolecule, τ_0 the average lifetime of the biomolecule, [Q] is the quencher concentration, and K_{sv} is the Stern–Volmer constant
$$log\frac{F_0-F}{F} = logK_b + nlog[Q]\ (3)$$
**Eq.3**<br>where F_0 and F are the steady-state fluorescence intensities at the maximum wavelength in the absence and presence of quencher, respectively, [Q] is the quencher concentration
$$logK_b = -\frac{\Delta H^{\circ}}{RT} + \frac{\Delta S^{\circ}}{R}\ (4)$$ $$\Delta G^{\circ} = \Delta H^{\circ} - T\Delta S^{\circ} = -RTlnK_b\ (5)$$
**Eq.4 and eq. 5**<br>where K_b is the binding constant, R is the universal gas constant, entropic change (ΔS°), free energy change (ΔG°), enthalpy change (ΔH°)
